# Micro‐Texturized and Ultra‐Soft Dry Electrode for Motion‐Artifact Tolerant and Long‐Term Stable Wearable Electrophysiological Monitoring

**DOI:** 10.1002/smll.202514966

**Published:** 2026-03-18

**Authors:** Sang‐Min Kim, Hee Jeong Jang, Ki‐Hoon Kim, Yeon‐Jeong Hwang, Yu Bin Lee, Joonsoo Jeong, Sunghoon Jung, Eubin Jeong, Dong‐Wook Han, Eui‐Suk Sung, Min‐Ho Seo

**Affiliations:** ^1^ Department of Information Convergence Engineering College of Information & Biomedical Engineering Pusan National University Busan Republic of Korea; ^2^ Department of Cogno‐Mechatronics Engineering College of Nanoscience & Nanotechnology Pusan National University Busan Republic of Korea; ^3^ School of Biomedical Convergence Engineering College of Information & Biomedical Engineering Pusan National University Yangsan Republic of Korea; ^4^ HUINNO Co., Ltd Seoul Republic of Korea; ^5^ Department of Otorhinolaryngology‐Head and Neck Surgery School of Medicine Pusan National University Yangsan Republic of Korea; ^6^ Research Institute for Convergence of Biomedical Science and Technology Pusan National University Yangsan Hospital Yangsan Republic of Korea

**Keywords:** biocompatible materials, carbon black composites, dry electrodes, electrophysiological monitoring, motion artifacts, skin adhesion, wearable sensors

## Abstract

Accurate and robust acquisition of electrophysiological signals is essential for wearable healthcare and human–machine interface applications. However, conventional wet electrodes are limited by motion artifacts and insufficient long‐term stability, while many dry electrodes lack adequate skin adhesion and mechanical compliance. Here, we present a carbon black (CB)–polydimethylsiloxane (PDMS) composite‐based dry electrode featuring a micro‐textured surface (R_a_ ≈ 1 µm), low modulus (∼216 kPa), and strong adhesive strength (∼15.68 kPa), conformally integrated onto a stretchable metallic interconnector with an optimized serpentine geometry (235° curvature angle). This architecture achieves high performance without fabrication complexity by leveraging spontaneously formed micro‐textures to promote mechanical interlocking for robust skin adhesion, thereby ensuring low interfacial impedance (∼165.2 kΩ·cm^2^ at 100 Hz) and enabling long‐term electrical and mechanical stability. To demonstrate real‐world applicability, we developed a wireless, miniaturized electrocardiogram (ECG) system integrated with an inertial measurement unit (IMU). The system achieved significantly higher signal‐to‐noise ratios (SNR) than commercial devices across diverse activities (16.82–26.19 dB vs. 4.98–13.8 dB) and maintained an SNR of 21.07 dB after 24 h of continuous monitoring. These results highlight the potential of the proposed electrode system as a scalable and practical solution for high‐fidelity, long‐term biosignal acquisition in wearable electronics.

## Graphical abstract

A highly conformal and adhesive dry electrode is developed using a micro‐textured carbon black/PDMS composite. By leveraging spontaneous surface roughness for enhanced mechanical interlocking and integrating a stretchable serpentine interconnector, the device achieves robust skin contact and low impedance. This simple, scalable approach enables long‐term stable electrophysiological monitoring under dynamic conditions.

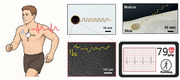

## 1 Introduction

The measurement of electrophysiological signals has gained significant importance in various applications, including disease diagnosis, healthcare, robotics, and human‐machine interfaces (HMI) [1, 2, 3, 4]. These technologies are essential not only for monitoring health status and evaluating therapeutic outcomes in real time but also for rapidly transmitting human intentions to machine systems. In this regard, accurate and real‐time acquisition of electrophysiological signals is a crucial component for next‐generation smart technologies, particularly in advanced healthcare systems and AI‐driven interfaces [5, 6, 7].

To achieve precise and stable acquisition of electrophysiological signals, various technological approaches have been explored [6, 8, 9]. Key advancements include low‐noise amplifier integrated circuits (LNA ICs), low‐power wireless communication systems, and flexible interconnection and packaging technologies [10, 11, 12]. These innovations have significantly enhanced the reliability and performance of electronic measurement hardware while improving user convenience, thereby accelerating the commercialization of medical devices and wearable systems [13].

Despite these advancements in hardware, electrode technologies have received relatively limited research attention, which has resulted in persistent difficulties in achieving stable and accurate electrophysiological signal measurements under active, real‐world wearable conditions. Conventional Ag/AgCl‐based gel‐coated wet electrodes offer high initial electrical conductivity; however, their rigid metallic snap components induce motion artifacts during user movement, compromising signal stability [14, 15]. Furthermore, the gradual evaporation of the gel over time leads to a rapid degradation of electrical properties, limiting their long‐term usability [6]. To address the limitations of conventional wet electrodes, semi‐dry electrodes have recently emerged as a promising intermediate category [16, 17, 18]. These systems typically employ localized electrolyte reservoirs or hydrogel interlayers to reduce interfacial impedance while maintaining certain practical advantages of dry electrodes. However, potential challenges such as electrolyte depletion during extended monitoring and increased structural complexity in some configurations motivate continued development of robust, fully dry sensing strategies.

To overcome these challenges, dry electrodes based on metallic thin films have been developed, offering improved mechanical flexibility and long‐term stability in dry environments [7, 19, 20]. Building on these solid‐state approaches, recent advancements have introduced soft electrodes based on metal nanowires, such as silver or copper nanowires [21, 22, 23, 24, 25], and liquid metals [26, 27, 28]. These materials offer superior mechanical compliance and conformability compared to traditional thin films, enabling more stable contact with the skin. However, these electrodes often exhibit insufficient skin adhesion force and contact, leading to unstable signal acquisition under dynamic and motion conditions [29, 30]. Recent developments in micro‐/nano‐structured electrodes aim to enhance skin contact through features such as micro‐needles or textured surfaces, effectively mitigating motion artifacts [31, 32, 33, 34]. However, these approaches involve complex fabrication processes, such as photolithography and etching, which significantly increase manufacturing costs and hinder scalability [35, 36, 37].

In this context, carbon‐based conductive composite dry electrodes have emerged as a promising alternative for motion‐artifact‐tolerant and long‐term stable bioelectrical signal acquisition with high simplicity and cost‐effectiveness. Microscopic carbon‐based particles such as graphene, carbon nanotubes (CNTs), and carbon black (CB) exhibit high electrical conductivity and excellent biocompatibility, while also providing mechanical softness when integrated with polymers such as polydimethylsiloxane (PDMS) [38, 39, 40]. Additionally, the ease of fabrication applicable to these materials, such as screen‐printing techniques, makes them attractive for addressing the limitations of traditional noble metal electrodes [41, 42, 43].

However, despite these possibilities, carbon‐based composite electrodes still suffer from significant limitations in terms of impedance, adhesion, and interconnection methods for reliable electrophysiological signal acquisition. Although carbon‐composite electrodes inherently contain electrically conductive carbon materials, they often exhibit higher impedance than required for high‐quality biosignal acquisition, necessitating further improvements to achieve lower impedance. In addition, from the perspective of adhesion, stable contact between the electrode and skin is critical for maintaining signal quality [6, 44]. However, simple carbon‐composite electrodes are fundamentally limited by insufficient contact force, with even slight variations in the carbon‐to‐polymer ratio or fabrication process causing substantial contact instability, thereby undermining reproducibility and signal reliability [45]. Moreover, the heterogeneous interface between carbon‐based composite electrodes and conventional metallic interconnectors remains a major source of electrical and mechanical instability, leading to increased contact impedance, signal loss, noise, and potential mechanical failure [46]. Nevertheless, traditional rigid metal snap interconnections or conductive ink‐based connections are still commonly used, limiting the inherent advantages of flexibility and stretchability offered by carbon‐based dry electrodes [42].

Herein, we propose a carbon black (CB)‐based dry electrode that is intrinsically tolerant to motion artifacts, exhibits low impedance, and remains fully compatible with conventional electrophysiological measurement systems. By leveraging the natural aggregation of CB particles during a simple screen‐printing process, we realized a micro‐texturized surface that enhances skin adhesion and lowers impedance. This approach stands in contrast to complex lithography‐based methods, demonstrating high performance without fabrication complexity. The central concept of the proposed approach lies in the conformal integration of an ultra‐soft and micro‐texturized CB‐based electrode material with a structurally optimized stretchable metallic interconnect, designed for enhanced mechanical compliance and reliability. Specifically, the ultra‐soft micro‐texturized surface, naturally formed on the conventional metallic interconnect through a simple screen‐printing process, significantly enhances skin adhesion while simultaneously reducing electrode‐skin impedance by increasing frictional force and effective surface area, respectively. In practical demonstration, the developed electrode exhibited strong and stable adhesion to both artificial skin substrates and real human skin, and consistently enabled reliable electrophysiological signal acquisition under a wide range of dynamic motion conditions, including variations in acceleration and movement frequency. To further validate its real‐world applicability, we implemented a wearable, wireless, motion‐detectable electrocardiogram (ECG) sensor system utilizing the developed electrode. Notably, the system achieved a consistently higher SNR than a commercial and conventional ECG system for over 24 h, even under active motion conditions, including walking, stretching, and running.

## 2 Results and Discussion

### 2.1 Overall Configuration and Interfacial Mechanics of the CB/PDMS Dry Electrode

**FIGURE 1:**
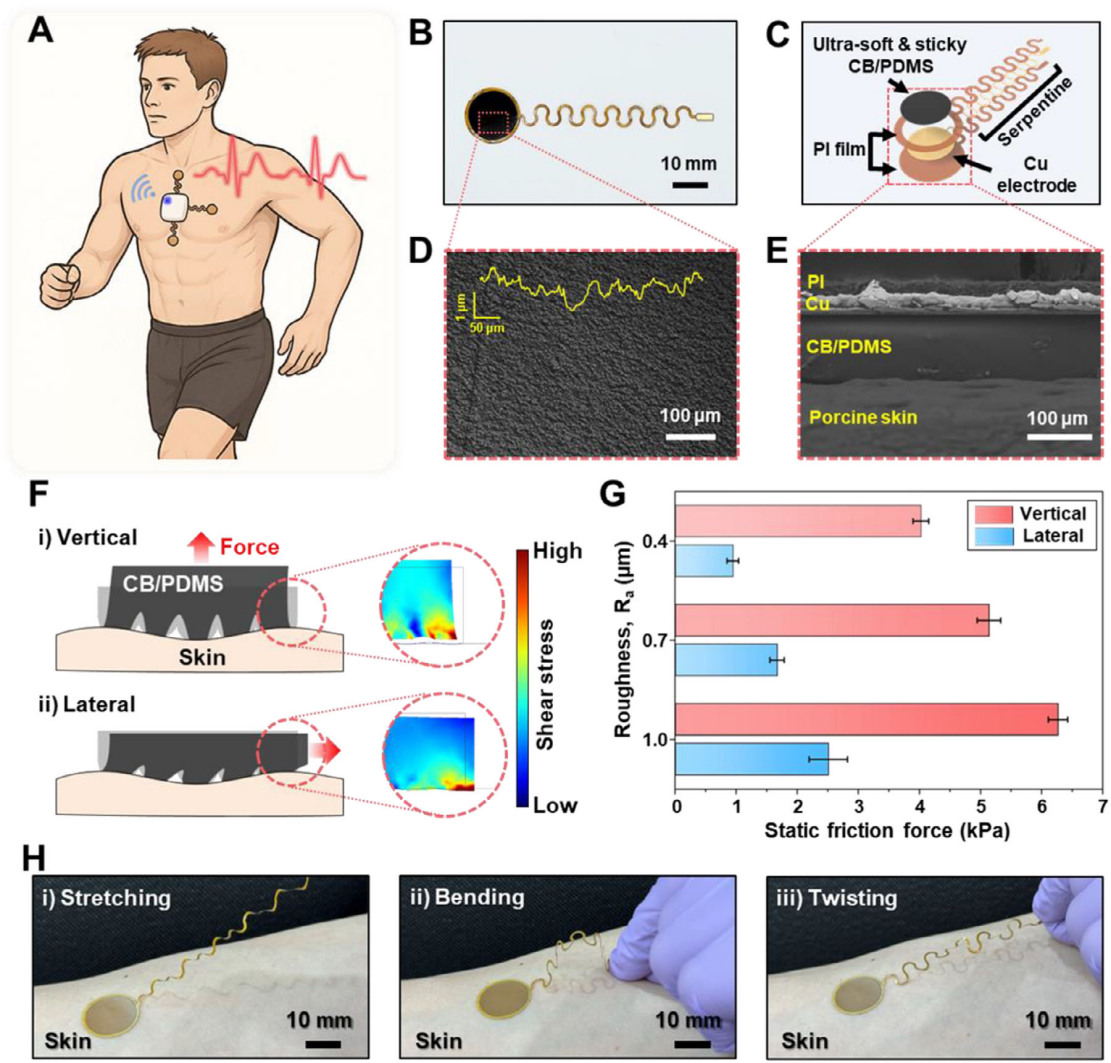
Configuration and interfacial mechanics of CB/PDMS composite dry electrodes with stretchable interconnectors. (A) Illustration of the wearable monitoring scenario showing the placement of the CB/PDMS dry electrode system on the human chest for stable biopotential acquisition during dynamic activities. (B) Optical photograph of the fabricated flexible dry electrode. (C) Cross‐sectional structure diagram showing the insulator‐metal‐insulator configuration of the serpentine interconnector. (D) 3D surface profile image revealing micro‐nano surface structures with R_a_ ≈ 1 µm achieved through optimized CB/PDMS composition. (E) Cross‐sectional SEM image demonstrating the conformal interface between the CB/PDMS electrode material and serpentine metal interconnector, with enhanced surface and structural stability. (F) Schematic illustration and corresponding FEM‐derived shear stress maps showing the interfacial stress distribution at the micro‐textured CB/PDMS–skin interface under vertical and lateral loading conditions. (G) Quantitative interfacial mechanics analysis showing the static friction forces generated under vertical and horizontal loading as a function of electrode surface roughness, demonstrating that increased micro‐texture reinforces frictional anchoring and reduces susceptibility to shear‐induced detachment. (H) Demonstration of skin adhesion characteristics during various mechanical deformations, including stretching bending, and twisting on human skin.

Figure 1A schematically illustrates the wearable use scenario, highlighting the placement of the electrode system on the human chest for stable biopotential acquisition during dynamic activities. An optical photograph of the fabricated flexible electrode is shown in Figure 1B. As depicted in Figure 1C, the CB/PDMS dry electrode is conformally integrated onto a polyimide (PI)–encapsulated copper (Cu) interconnect with a serpentine geometry. Detailed fabrication procedures are provided Figure S1 and the Experimental Section. Notably, the CB/PDMS composite naturally forms 3D micro‐topographies (R_a_ ≈ 1 µm) without the need for sophisticated microfabrication processes, owing to the aggregation of nanoscale carbon black (Vulcan XC 72R, Fuel Cell Store, USA) particles within the polymer matrix (Sylgard 184, Dow Corning, USA), as shown in Figure 1D. Furthermore, the developed CB/PDMS composite exhibits a significantly lower mechanical modulus than that of human skin, enabling intimate and conformal contact at the electrode–skin interface (Figure 1E).

The developed electrode significantly enhances skin adhesion owing to its micro‐scale surface texturing and low mechanical modulus. Figure 1F schematically illustrates the conceptual deformation modes and the corresponding shear‐stress distributions localized at the highlighted skin–electrode interface. Under both (i) vertical and (ii) lateral loading conditions, the developed electrode exhibits a mechanical modulus more than an order of magnitude lower than that of skin. As a result, lateral deformation of the electrode occurs regardless of the loading direction, effectively inducing substantial shear stress at the skin–electrode interface (COMSOL finite element method (FEM) simulation results are shown in Figure 1F). Importantly, under such shear‐dominant conditions, the micro‐texturized electrode with micrometer‐scale asperities exhibits enhanced frictional resistance that suppresses interfacial sliding. This behavior is consistent with Persson's contact mechanics framework, which predicts that for elastically deformable solids under conformal contact, increased surface roughness enhances multi‐asperity engagement and interfacial shear stress, thereby increasing macroscopic friction [47]. Building on these mechanics‐based insights, the COMSOL FEM calculation provides quantitative verification that increased surface roughness enhances resistance to shear‐induced detachment (Figure 1G). As the micro‐texture becomes more pronounced, the static friction force measured under both vertical and horizontal loading conditions increases substantially, confirming that rougher surfaces promote stronger frictional anchoring at the skin–electrode boundary. These results support the conclusion that the micro‐asperities not only influence the distribution of shear stress (Figure 1F) but also contribute to improved resistance against shear‐driven detachment during motion. Detailed information regarding the FEM simulation is provided in the Experimental Section. In practice, the developed electrode maintains robust adhesion to the skin under practical deformation modes, such as stretching, bending, and twisting, without noticeable delamination (Figure 1H). This experimentally observed stability originates from the low modulus of the CB/PDMS composite and its micro‐textured surface, which together enable strong frictional coupling and sustained conformal contact with the skin during motion.

### 2.2 Material Properties Optimization and Characterization of Micro‐Textured CB/PDMS Conductive Composite

The optimized 3D microstructure enhances adhesion to human skin through systematic control of the PDMS‐to‐CB blend ratio. Unlike conventional dry electrodes based on conductive particle composites, which simply target electrical conductivity enhancement within the saturation region beyond the percolation threshold, this study investigates the influence of conductive particle concentration on the electrode's physical structure after electrical property saturation. According to percolation theory, electrical conductivity increases rapidly when CB concentration exceeds the threshold value, but this improvement plateaus beyond a certain concentration. This plateau behavior is consistent with classical percolation theory, which predicts that once a continuous conductive network is established, further addition of conductive fillers results in diminishing improvements in macroscopic conductivity [48]. Beyond the percolation threshold, conductive pathways are already sufficiently interconnected, and the overall conductivity becomes increasingly governed by inter‐particle tunneling and contact resistance rather than by filler concentration itself [49]. Consequently, additional CB particles contribute marginally to conductivity enhancement while significantly influencing the composite microstructure. At higher filler loadings, intensified particle–particle interactions promote aggregation of nano‐sized CB domains within the PDMS matrix [50]. Such aggregation progressively manifests as micrometer‐scale surface asperities, resulting in spontaneous microstructure formation on the electrode surface without additional fabrication steps. From a contact mechanics perspective, when a compliant elastomer with surface asperities contacts a comparatively stiffer substrate, deformation is preferentially accommodated within the softer material, enabling conformal engagement of surface features and enhanced interfacial shear resistance. According to Persson's contact mechanics theory, the frictional force between elastic solids increases with surface roughness under conformal contact conditions due to multi‐asperity interactions and distributed shear stress across the interface [47]. Consistent with this framework, our finite element simulations (Figure S2) demonstrate that deformation is predominantly localized within the softer electrode layer, promoting asperity engagement at the interface and increasing resistance to shear‐induced sliding.

Based on this contact‐mechanics framework, we hypothesized that the aggregation‐induced microscale surface features enhance mechanical interlocking with the skin, thereby improving adhesion strength.

In essence, this structural evolution increases surface roughness without introducing fabrication complexity, allowing mechanical adhesion to be enhanced while the composite remains within a fully percolated conductive regime.

**FIGURE 2:**
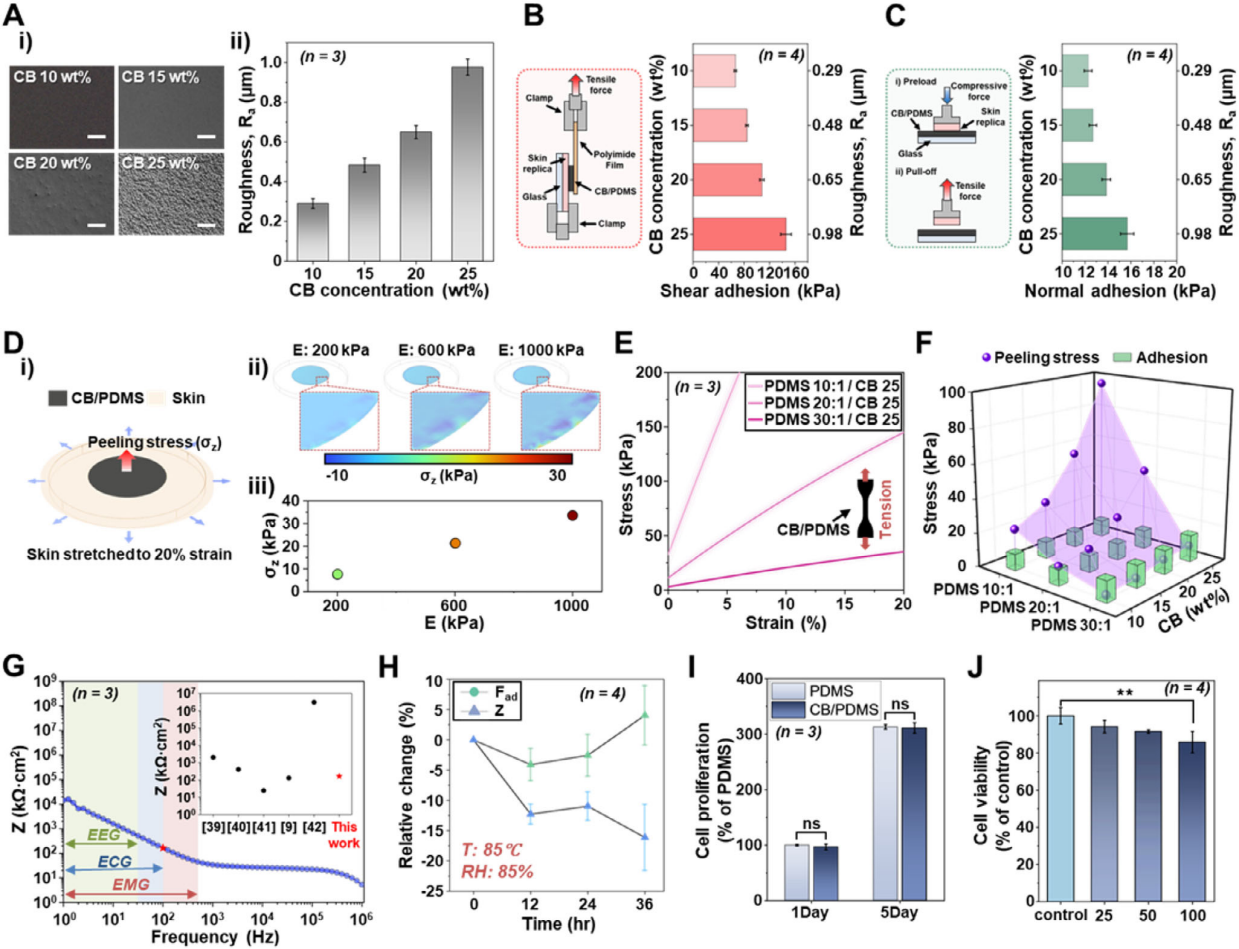
Material optimization and characterization of micro‐structured CB/PDMS conductive composites. (A) 3D optical surface images (scale bars: 50 µm) and profiles obtained using laser‐based optical imaging (VHX‐7100, Keyence) showing surface morphology evolution with increasing CB concentration (10, 15, 20, and 25 wt% in PDMS, *n = 3*). (B) Schematic of the shear‐direction (180°) adhesion test and corresponding adhesion strength variation with CB concentration (surface roughness) (*n = 4*). (C) Normal‐direction adhesion strength as a function of CB concentration, showing enhanced interfacial adhesion with increased CB content (*n = 4*). (D) Finite element method (FEM) analysis of peeling stress (σ_z_) as a function of electrode stiffness under 20% skin tension. (E, F) Experimental stiffness measurements (*n = 3*) and calculated peeling stresses for different electrode compositions, demonstrating mechanically optimized conditions at 30:1 PDMS with 25 wt% CB. (G) Electrochemical impedance spectroscopy (1–10^6^ Hz) showing impedance characteristics of optimized electrodes (165.2 kΩ·cm^2^ at 100 Hz). (H) Long‐term stability evaluation under accelerated aging conditions (85°C/85% RH) showing changes in adhesive strength and electrical impedance over 36 h. (I, J) Biocompatibility assessment using L‐929 cells: (I) cell proliferation test over 5 days (*n = 3*) and (J) extract cytotoxicity test showing >80% cell viability compared to controls (*n = 4*) (ns: not significant, ^**^ *p* < 0.01 vs. control).

To validate this hypothesis, 4 different composite dry electrodes were fabricated with CB concentrations of 10, 15, 20, and 25 wt% in PDMS, respectively, and their surface morphologies were characterized using a digital microscope (VHX‐7100, Keyence, USA) (Figure 2A‐i). The measured optical results demonstrate that surface roughness increases markedly with CB concentration (Figure 2A‐ii). For quantitative analysis, surface structures were examined using laser‐based optical imaging (VHX‐7100, Keyence, USA). Roughness measurements extracted from the acquired images revealed that CB precipitation on the surface increases with concentration, resulting in surface roughness increases from hundreds of nanometers to micrometers (Figure S3). Specifically, the electrodes exhibiting surface roughness (R_a_) of approximately 300 nm at 10 wt% CB showed a continuous increase in roughness with increasing CB concentration, reaching approximately 1 µm at 25 wt%. While greater roughness could theoretically further enhance frictional anchoring, increasing the CB concentration beyond 25 wt% resulted in incomplete curing due to inhibition of the hydrosilylation reaction and severe processing instability arising from excessive viscosity [51, 52]. Therefore, the 25 wt% composition was identified as the practical optimum, representing the highest attainable surface roughness while maintaining mechanical integrity and reliable processability of the composite.

To elucidate the microstructural origin of this roughness evolution, cross‐sectional SEM analysis was performed for CB/PDMS composites with varying CB concentrations (Figure S4). At lower CB loadings (10 and 15 wt%), CB particles were relatively well dispersed within the PDMS matrix, forming isolated domains. In contrast, higher CB contents (20 and 25 wt%) exhibited pronounced CB‐rich clusters and interconnected domains across the composite cross‐section. Quantitative image analysis using ImageJ revealed a monotonic increase in the projected area of CB‐rich regions with increasing CB concentration, confirming progressive aggregation within the matrix. These results directly support that increased CB loading promotes micro‐scale structural heterogeneity, which contributes to surface roughness evolution and enhanced mechanical interlocking.

To assess the direct impact of enhanced surface roughness on adhesive strength, electrodes with different compositions were tested against commercial artificial skin (Suturing Skin Pad, HertzHardware, China; elastic modulus ≈ 1.02 MPa) using mechanical adhesion measurements under both shear and normal directions. An adhesive strength measurement system was constructed using an automated push‐pull gauge (M5‐2 & ESM303, Mark‐10, USA) (Figure S5). For the shear‐direction (180°) adhesion test, the CB/PDMS electrode laminated on a polyimide (PI) film was attached to the artificial skin, and the assembly was peeled off at a 180° direction using a mechanical tester to simulate realistic sliding motion at the electrode–skin interface (Figure 2B). The maximum detachment force recorded during peeling was divided by the initial contact area to calculate the shear‐adhesion strength. The measured values exhibited a clear increasing trend with CB concentration, confirming that higher CB content enhances interfacial friction and mechanical interlocking through progressively formed surface micro‐textures. For the normal‐direction adhesion test, silicon‐based artificial skin was mounted on the system tip, a specific pressure (11.1 kPa) was first applied to the electrode, and the force measured during contact release was defined as the adhesive strength during gradual artificial skin tensioning. The results (Figure 2C) showed a consistent monotonic increase in adhesion strength with increasing CB concentration, supporting the conclusion that surface micro‐roughness rather than the test orientation governs the adhesion enhancement. In addition, the time‐dependent adhesion stability of the optimized CB/PDMS composite was examined to verify potential variation in adhesive performance over time. Adhesion tests conducted immediately after fabrication and after 24 h of storage showed no degradation, with shear‐adhesion strength remaining stable (17.16 ± 0.66 kPa → 18.37 ± 1.14 kPa) (Figure S6). These results confirm that the optimized composite formulation maintains reliable self‐adhesion even after extended storage, supporting its suitability for long‐term wearable applications. To further investigate the combined effects of composite composition and matrix stiffness on adhesion behavior, electrodes with various PDMS base‐to‐curing agent ratios (10:1, 20:1, and 30:1) and CB concentrations (10–25 wt% in PDMS) were systematically evaluated. As shown Figure S7, adhesive strength increased with higher PDMS base ratios due to the enhanced viscoelasticity and softness of the polymer matrix, which promoted conformal contact with the skin. Moreover, at each PDMS ratio, increasing CB content further improved adhesion owing to the formation of microscale surface textures that enhance interfacial friction and mechanical interlocking. These results demonstrate that both polymer network softness and filler‐induced surface roughness synergistically contribute to maximizing the overall adhesion performance of the CB/PDMS composite electrodes. Compared with previously reported bio‐inspired or micro‐structured dry electrodes, the developed micro‐textured CB/PDMS electrode demonstrates excellent adhesion in both normal and shear directions, maintaining reliable and repeatable skin contact under motion (Table S1).

Beyond adhesive strength, mechanical stiffness represents a critical factor for reliable skin contact. Electrodes with stiffness exceeding that of skin may fail to accommodate skin deformation during activity, creating a mechanical mismatch that leads to electrode detachment. To address this consideration, finite element method (FEM) analysis examined peeling stress during activity as a function of electrode stiffness. In the FEM simulation, we calculated the induced peeling stress (σ_z_) in the electrode by 20% of skin‐tension, which is a typical deformation in various body activities, as a function of the electrode stiffness (Figure 2D; Figure S8). The results indicated that peeling stress decreased with reduced electrode stiffness, with MPa‐range stiffness electrodes exhibiting peel forces exceeding 30 kPa, while stiffness reduction to 200 kPa decreased peeling stress below 10 kPa.

Based on FEM results, experimental stiffness measurements were further conducted for electrodes of different compositions. For the experiment, dog‐bone‐shaped electrode specimens were subjected to tensile testing, and stiffness at 20% strain was extracted (Figure S9). Using measured stiffness values, electrode peeling stresses under skin tensile conditions (20%) were calculated, revealing that electrode stiffness increased with lower base ratios and higher CB concentrations, with corresponding peel force increases (Figure 2E,F). Specifically, electrodes with base‐to‐curing agent ratios of 10:1 or 20:1 exhibited skin‐electrode peeling stresses of 9.36–96.16 kPa across all CB concentrations, significantly exceeding skin‐electrode adhesive strength and indicating difficulty in maintaining stable contact by skin deformation. However, at a sufficiently low curing agent ratio (30:1), peeling stress drastically decreased below 10 kPa. Notably, the composite with 25 wt% CB in 30:1 PDMS exhibited a peeling stress of only 8.05 kPa, approximately 50% of the electrode's intrinsic adhesive strength (15.68 kPa), indicating mechanically optimized fabrication conditions capable of maintaining stable contact even under skin deformation. Through systematic studies on adhesive strength and deformation‐induced peeling behavior, the CB/PDMS electrode composed of PDMS with a 30:1 base‐to‐curing agent ratio and 25 wt% CB was identified as the optimal formulation for achieving both strong skin adhesion and mechanical compatibility.

Electrical impedance properties are equally important for bioelectrode applications. Therefore, impedance characteristics of electrodes with optimized mechanical contact properties (30:1 PDMS with 25 wt% CB) were investigated. Fabricated electrodes were immersed in phosphate buffer solution (pH 7.4) and subjected to electrochemical impedance spectroscopy (EIS) from 1 to 10^6^ Hz (Figure 2G). EIS measurements were conducted in phosphate‐buffered saline (PBS, pH 7.4) to mimic physiological ionic conditions at the skin–electrode interface, as commonly adopted in prior biopotential electrode studies [53, 54, 55]. Results showed continuously decreasing impedance from 1 to approximately 500 Hz, remaining constant at higher frequencies, indicating ideal metallic properties suitable for stable bioelectrical signal measurement. The impedance at 100 Hz was 165.2 kΩ·cm^2^, lower than the impedance of previously developed dry electrodes (inset in Figure 2G) [9, 56, 57, 58, 59], confirming that the developed electrode can reliably measure bioelectrical signals while maintaining relatively low interfacial impedance. Electrical impedance data for all fabrication conditions are provided Figure S10.

Beyond impedance magnitude, electrochemical baseline stability is essential for reliable long‐term biopotential recording. To evaluate DC stability, open‐circuit potential (OCP) measurements were conducted in PBS (pH 7.4) for 10 min (Figure S11). The developed electrode exhibited an equilibrium DC offset potential of 20.89 mV, which satisfies the ANSI/AAMI EC12:2000/(R)2020 requirement (<100 mV) for disposable ECG electrodes. The potential drift, determined from the linear slope of the OCP–time curve, was 0.946 µV/s, indicating limited baseline variation over the 10 min measurement period. Together with the impedance characteristics, these results demonstrate that the developed CB/PDMS dry electrode provides electrically stable behavior under physiologically relevant ionic conditions.

To further evaluate long‐term reliability under accelerated conditions, the stability of the optimized electrodes was subsequently assessed through an accelerated environmental test. Optimized electrodes (30:1 PDMS with 25 wt% CB) were stored at 85°C/85% RH for 36 h, and changes in adhesive strength and electrical impedance were measured (Figure 2H).

The adhesive strength initially decreased by 3.98% at 12 h, followed by a gradual recovery, ultimately exceeding the baseline by 3.51% after 36 h. To elucidate the underlying mechanism, Raman spectroscopy was performed on aged samples (Figure S12), revealing a substantial reduction (80.9%) in the Si─H stretching peak intensity. This indicates consumption of residual hydrosilane (Si─H) groups and additional crosslink formation during hygrothermal exposure. Previous studies have reported that residual Si─H groups can undergo secondary reactions under thermal and humid conditions, forming additional Si─O─Si linkages and increasing network density [60]. Furthermore, hygrothermal aging of silicone materials has been shown to promote structural stabilization through post‐curing and network rearrangement [61].

To further examine surface chemical changes, static water contact angle measurements were conducted as a function of aging time (Figure S13). A gradual decrease in contact angle was observed after prolonged exposure, indicating enhanced surface polarity. This trend suggests increased formation of polar silanol groups and moisture‐assisted surface modification, which can facilitate interfacial hydrogen bonding interactions [33]. The combined effects of network stabilization and increased surface polarity contribute to the recovery and stabilization of adhesion during aging.

The impedance at 100 Hz decreased continuously by 12.53% at 12 h and 16.87% after 36 h. PDMS contains low‐molecular‐weight siloxane oligomers and residual volatile species that can diffuse and evaporate during high‐temperature post‐curing [62]. The removal of these mobile species reduces microstructural inhomogeneity and enhances dielectric stability by forming a more uniform crosslinked network. Therefore, the observed impedance reduction is attributed to synergistic effects of additional network stabilization and reduced compositional heterogeneity within the composite matrix. These results demonstrate that the developed electrodes maintain or even improve their mechanical and electrical properties under harsh conditions, confirming their suitability for long‐term wearable bioelectronic applications. Furthermore, to assess long‐term usability, the adhesion durability of the optimized electrode was evaluated under repeated peel–reattach cycles. As shown Figure S14, the adhesion strength slightly decreased from 13.32 to 10.09 kPa after 10,000 cycles (∼24% reduction) but remained above the design threshold (8.05 kPa), demonstrating excellent reusability and long‐term stability suitable for continuous wearable operation.

To further assess suitability for long‐term application, biocompatibility was evaluated using L‐929 cells. In cell proliferation tests (Figure 2I), specimens of 10 mm diameter and 100 µm thickness were prepared and monitored for 5 days. Cell proliferation rates were similar to PDMS (10:1) controls, and cell viability exceeded 80% compared to controls (TCP) in extract cytotoxicity tests (Figure 2J), confirming excellent biocompatibility.

In addition to the extract‐based cytotoxicity evaluation, a preliminary human skin compatibility test was conducted to assess direct dermal contact safety. Four electrodes were simultaneously attached to the forearm and sequentially removed at 6 h intervals up to 24 h. No visible signs of erythema, edema, or irritation were observed upon removal, and temporary indentation marks fully recovered within 1 h (Figure S15). Previous studies have reported the safe dermal application of CB/PDMS‐based adhesive electronics under repeated skin attachment conditions without observable irritation [63]. Considering the well‐established biocompatibility of PDMS and its widespread use in wearable and medical devices, these findings support the suitability of the developed dry electrode for prolonged and repeated skin contact under practical use conditions.

Overall, the composite electrode with a PDMS 30:1 and 25 wt% CB formulation exhibited optimal properties for long‐term bioelectronic applications, including strong adhesion (15.68 kPa), soft stiffness (216 kPa), stable and low electrical impedance (165.2 kΩ·cm^2^ at 100 Hz), long‐term environmental stability, and excellent biocompatibility.

### 2.3 Electrode Structure Optimization and Electro‐Mechanical Stability Evaluation

**FIGURE 3:**
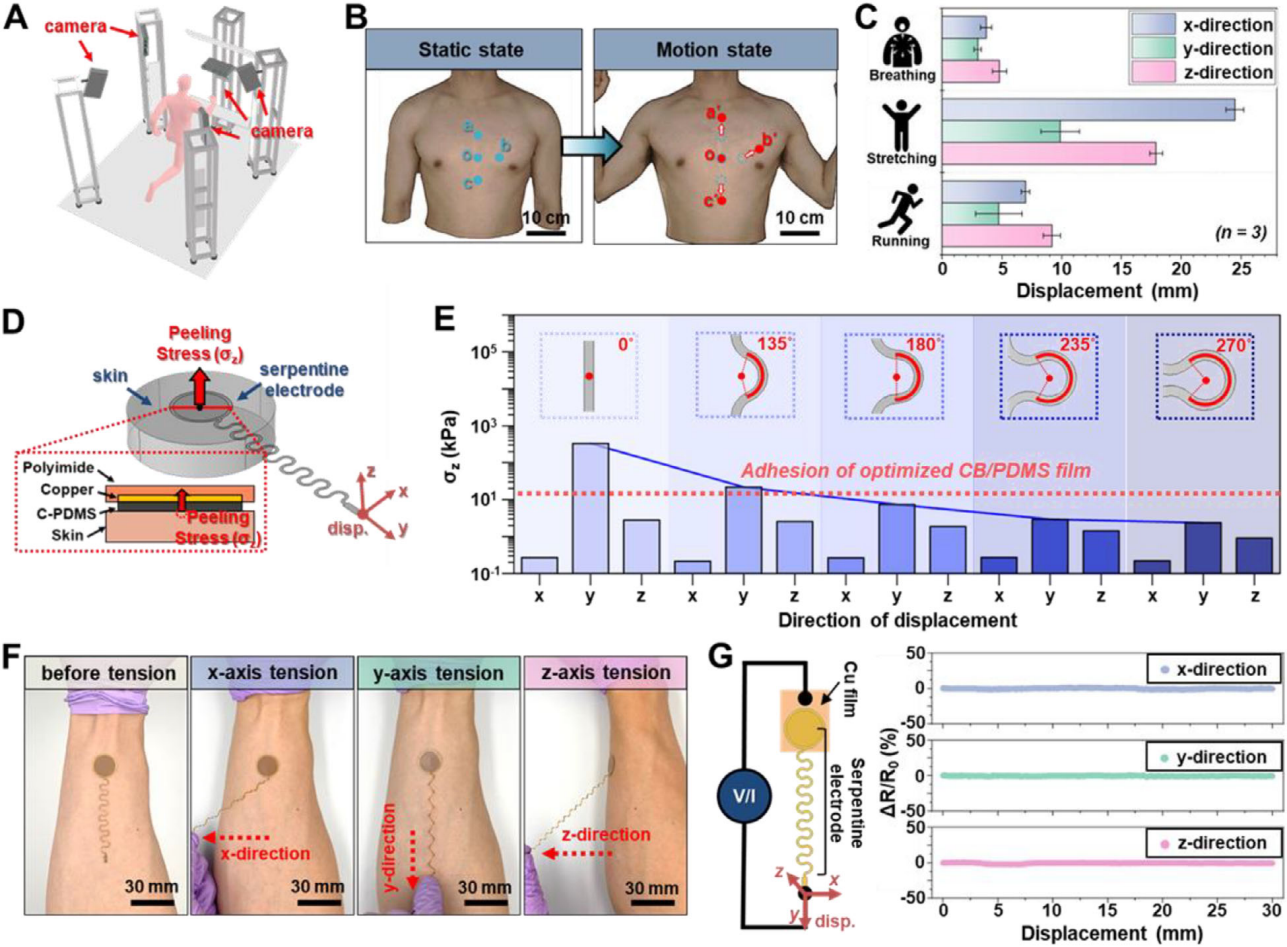
Electrode structure optimization and electro‐mechanical stability evaluation. (A) 3D motion capture system setup using five high‐speed cameras for quantitative skin deformation analysis. (B) Designated measurement locations (a, b, c, o) on the human body for electrode attachment sites. (C) Quantified skin surface displacements during daily activities (*n = 3*): breathing (4.8 ± 0.7 mm), running (8.7 ± 1.2 mm), and stretching (23.6 ± 2.3 mm). (D) Serpentine interconnector structure design with variable curvature angles for FEM simulation. (E) FEM simulation results showing peeling stress reduction in x, y, and z directions as serpentine angle increases, with optimal performance at 235° (peeling stress ≈ 2.9 kPa). (F) Experimental validation of electrode adhesion stability using optimized structure (235° serpentine angle) and material composition during >30 mm displacement in multiple directions. (G) Quantitative electro‐mechanical stability evaluation showing resistance changes during triaxial tensile testing for different serpentine angles, with optimized 235° structure maintaining <2% resistance variation under 30 mm displacement.

To realize a highly practical wearable system capable of reliable biosignal measurement under skin deformation caused by human body motion, not only material optimization but also optimization of the interconnection structure is essential. In this study, a conventional serpentine interconnect design was systematically optimized based on experimentally obtained upper‐body deformation data obtained from motion‐capture analysis. Motion‐capture analysis of realistic torso movements (multi‐axial extension) provided displacement and curvature fields representative of actual wear conditions. These were implemented in FEM to derive an interconnect geometry that preserves structural integrity and electrical continuity under in‐plane and out‐of‐plane deformation (Figure 3A–C). The optimized 235° serpentine design minimizes local stress concentration and enhances mechanical reliability under realistic motion scenarios, demonstrating a wear‐site‐specific optimization strategy for stretchable systems. First, to quantitatively measure physical skin deformation due to human motion in wearable environments, an optical motion capture system (3dMDBody, 3dMD LLC, USA) consisting of five high‐speed cameras was utilized (Figure 3A). Three adult male subjects were used to measure skin surface displacements in x, y, and z directions during everyday movements, including breathing, stretching, and running. Locations where electrodes and biosignal measurement systems would be physically attached were designated as a, b, c, and o (Figure 3B), and skin deformation at each location was quantified as 4.8 ± 0.7 mm for breathing, 8.7 ± 1.2 mm for running, and 23.6 ± 2.3 mm for stretching (Figure 3C). Detailed deformation visualizations for each activity are presented Figure S16.

Based on these motion displacement measurement data, FEM simulations were performed (Figure 3D). Since the serpentine structures provide structural advantages of distributing mechanical stresses on electrodes while allowing flexibility by structural curvature (serpentine angle), the angle was established as the primary design variable. This enabled modeling (using COMSOL Multiphysics) of peeling stress (σ_z_) induced at the skin‐electrode interface due to positional differences between electrodes (at a, b, and c) and wearable devices (at o) when relative electrode displacement occurs due to human body motion. It should be noted that for the FEM calculation, the mechanical properties of 25 wt% and 30:1 CB/PDMS electrodes were incorporated (Figure 2). Figure 3E presents simulation results for peeling stress in x, y, and z directions as the serpentine angle varies (0° to 270°). Under displacement conditions up to 30 mm, sufficient to accommodate the relative displacement at electrode attachment locations, peeling stress in each direction gradually decreases with increasing serpentine angle due to structural buffering effects. Particularly, when the serpentine angle is over 235°, peeling stress in all directions is reduced to 2.9 kPa or less, adequately accommodated by optimal electrode material adhesion strength (≈ 16 kPa in Figure 2), enabling stable signal measurement without peeling during actual use. Detailed simulation results are summarized Figure S17.

Actual adhesion stability of electrodes fabricated using optimal structure (serpentine angle 235°) and material composition (PDMS 30:1/CB 25 wt%) derived from simulations was experimentally evaluated (Figure 3F). By attaching electrodes to a human arm and subjecting them to displacements exceeding 30 mm in various directions without adhesion aids, electrodes demonstrate reliable skin adhesion. This result agrees well with simulation predictions and demonstrates the effectiveness of optimized structure and material composition.

For quantitative verification of electro‐mechanical stability, Figure 3G shows resistance changes during triaxial tensile testing using Mark‐10 equipment as the serpentine angle varies (Figure S18). The 0° structure exhibited resistance changes of 18.7% in the x‐direction (fracture after 19.38 mm), 26.8% in the y‐direction (fracture after 0.95 mm), and 3.95% in the z‐direction, with electrical stability improving in each direction as the serpentine angle increased (Figure S19). Notably, the optimized 235° structure showed negligible resistivity changes of 1.91, 2.07, and 2.29% in x, y, and z directions, respectively, maintaining stable electrical properties within 2% even under displacement conditions up to 30 mm. These results, combined with previously identified optimal material compositions, suggest the possibility of realizing highly reliable electrode systems capable of stable biosignal measurement under human motion.

### 2.4 Wearable System Implementation and Benchtop Performance Evaluation

**FIGURE 4:**
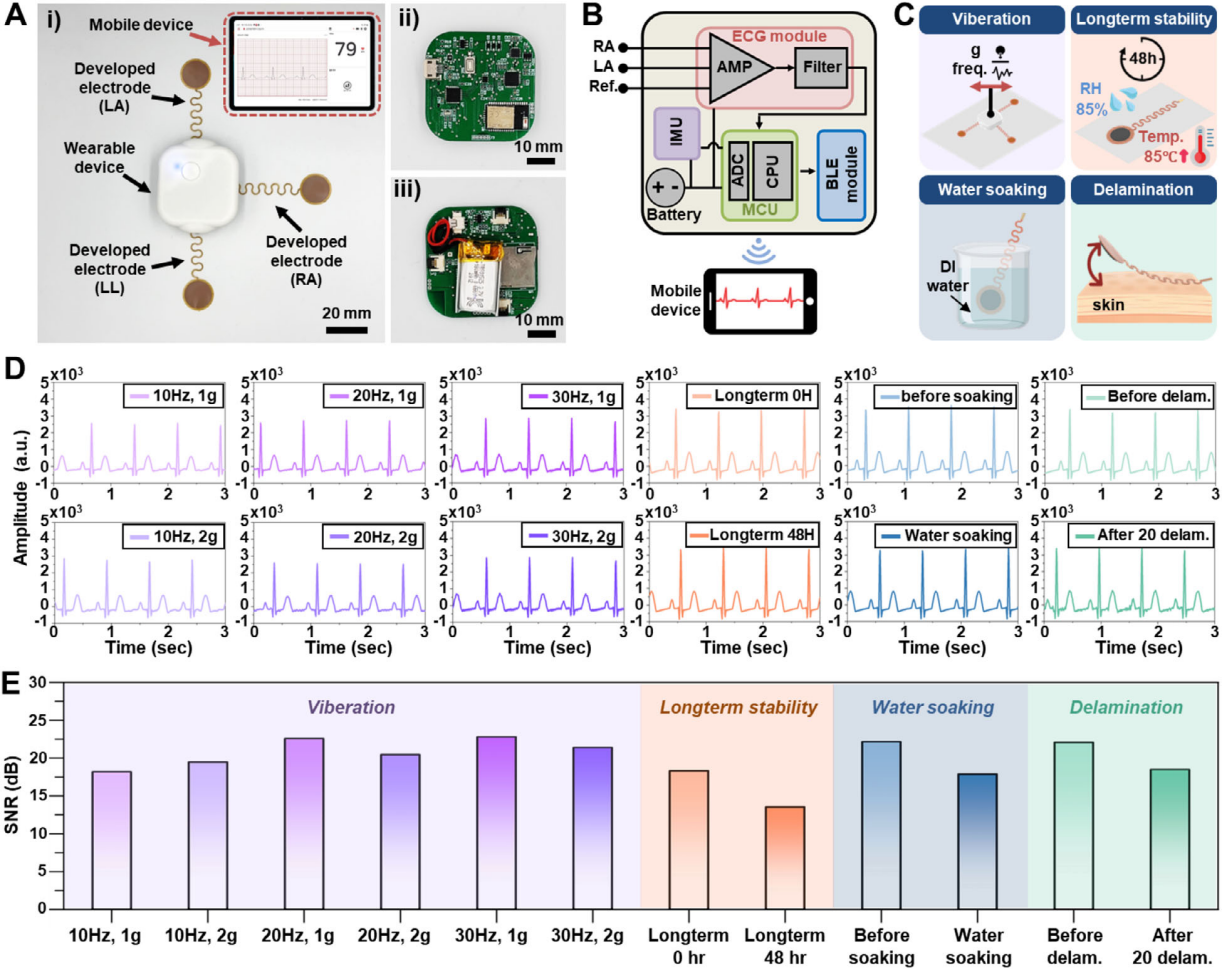
Wearable system implementation and performance verification under simulated real‐world conditions. (A) Miniaturized wireless biosignal monitoring device integrated with developed electrodes, showing a complete wearable system. (B) System operation flowchart illustrating signal acquisition, processing, and wireless transmission pathway from electrodes through ECG chip (MAX30003, Analog Devices Inc., USA), microcontroller unit (MCU, STM32L432KCU6, STMicroelectronics, Switzerland), to mobile device via Bluetooth, with simultaneous IMU‐based activity monitoring. (C) Four primary experimental conditions for performance evaluation: vibration testing (10–30 Hz, 1–2 g), long‐term stability (85°C/85% RH, 48 h), moisture resistance, and electrode reusability assessment. (D) Representative ECG waveforms showing maintained PQRST characteristics under all experimental conditions using ECG generator (MS400). E) Signal‐to‐noise ratio (SNR) analysis across experimental conditions: vibration testing (18–23 dB), long‐term stability (18→13 dB after 48 h), water resistance (22→18 dB), and reusability (22→18 dB after 20 cycles), all exceeding a minimum 5 dB threshold for reliable QRS detection.

Based on developed electrodes, a wireless wearable biosignal monitoring system was fabricated using commercially available chips and connected to the electrodes via a PI‐based flexible printed circuit board (FPCB), realizing a complete wireless wearable device whose performance was systematically verified under various real‐world conditions (Figure 4A–B; Figure S20). The details about the wearable measurement system are presented in the Experimental Section.

Using the developed instrumentation system, four primary experiments simulating real‐world conditions were conducted (Figure 4C). First, vibration testing evaluated motion artifact influence under frequency ranges (10–30 Hz) and acceleration conditions (1–2 g) reflecting daily human body movement. Second, long‐term biosignal measurement stability testing involved storing the system at 85°C and 85% humidity for 48 h to observe ECG measurement performance changes. Third, moisture stability testing evaluated moisture exposure effects by immersing electrodes in water, followed by removal. Finally, to confirm electrode reusability, the impact of dead skin cells on signal quality during repeated attachment and detachment was analyzed. To ensure fair and reliable performance comparison, the biosignal measurement capability of developed electrodes and systems was evaluated using an ECG generator (MS400 Multiparameter Simulator, CONTEC MEDICAL SYSTEMS, China).

Measurement results show that characteristic PQRST waveforms of ECG signals are clearly maintained under all experimental conditions (Figure 4D). Signal‐to‐Noise Ratio (SNR) analysis was performed to quantitatively evaluate ECG signal quality under each experimental condition. (Figure 4E). For vibration testing, SNR changes were analyzed as functions of frequency and acceleration. SNR values were approximately 18 dB at 10 Hz/1 g, 22 dB at 20 Hz/1 g, and 23 dB at 30 Hz/1 g, remaining stable at 19, 20, and 21 dB, respectively, even when acceleration increased to 2 g. In long‐term stability testing, SNR decreased slightly from an initial 18 to 13 dB after 48 h but remained at levels allowing main ECG signal feature identification. In water resistance testing, SNR decreased slightly from 22 dB before water contact to 18 dB after contact. In peel testing, SNR decreased from an initial 22 to 18 dB after 20 repetitions.

These evaluation results demonstrate that the developed wearable system maintains stable performance across various real‐world usage environments. Notably, SNR was maintained above 12 dB under all test conditions, significantly exceeding the minimum 5 dB standard required for reliable QRS waveform detection [64]. This demonstrates that the developed technology resists various external factors occurring in real‐world usage environments, including vibration, moisture exposure, and prolonged use, indicating its potential as a practical wearable healthcare device.

### 2.5 Applications for Wearable Systems: Measurement of Electrophysiological Signals

To verify the practical applicability of the developed wearable system, a comprehensive evaluation of ECG measurement performance during various daily activities was conducted using the developed device. System performance was systematically analyzed in terms of real‐time monitoring capability, activity status detection, and signal quality maintenance.

In this study, the ECG + IMU wireless configuration was not merely adopted as a conventional integration but was systematically optimized to enable quantitative motion–signal correlation analysis. While previous studies primarily utilized ECG and IMU sensors for activity classification or posture recognition, some studies have explored approaches for reducing motion artifacts; however, they exhibited relatively limited performance, showing lower signal‐to‐noise ratios (SNR) compared with commercial devices and improvements only under specific motion conditions.

Our system advances beyond previous approaches by performing IMU‐based multi‐parametric analysis of motion influence on ECG quality, including SNR, power spectral density (PSD), and signal‐stability indices (ST and TP segments) across various motion states (lying, sitting, walking, running). This integrated approach provides a quantitative framework for evaluating motion–signal interactions, offering practical insights for reliable physiological monitoring in dynamic, real‐world conditions.

**FIGURE 5:**
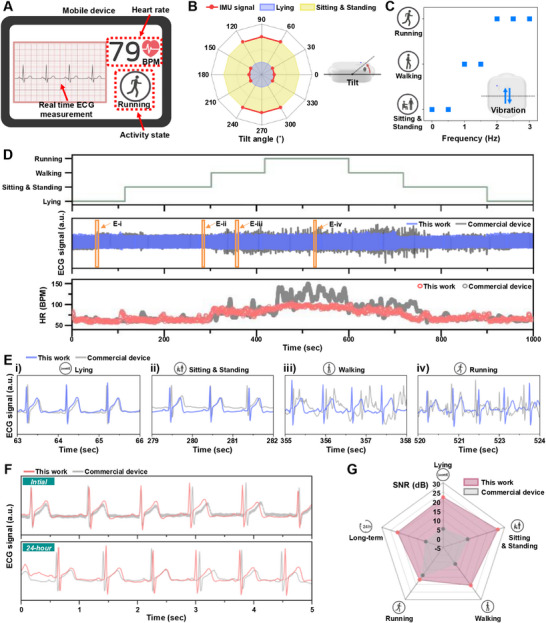
Real‐world application validation and performance comparison with commercial devices. (A) Mobile application interface displaying real‐time ECG waveforms, heart rate (BPM), and activity status via Bluetooth Low Energy (BLE) communication. (B) IMU sensor calibration for static posture classification showing tilt angle responses (0–360°) with reliable detection of lying down (330–30°, 150–210°) and sitting/standing positions (60–120°, 240–300°). (C) IMU frequency response characterization using vibration generator (0–3 Hz) for activity classification: sitting/standing (0–0.5 Hz), walking (1–1.5 Hz), and running (≥2 Hz). D) Comprehensive 1000‐s measurement comparing developed device (blue) with commercial device (MEMO Patch 2 with 2223H electrodes) (gray) across multiple activities, showing activity state tracking (top), ECG signals (middle), and heart rate trends (bottom). (E) Magnified ECG signal comparison during specific activities: (i) lying down, (ii) sitting/standing, (iii) walking, and (iv) running, demonstrating superior signal quality and PQRST waveform preservation of the developed device across all conditions. (F) Long‐term stability comparison showing ECG signal quality maintenance after 24‐h continuous measurement. (G) Radar chart presenting SNR analysis across all activities and long‐term measurement, with developed device achieving superior performance: lying (22.12 vs 4.98 dB), sitting/standing (26.19 vs 8.96 dB), walking (20.5 vs 6.18 dB), running (16.82 vs 13.8 dB), and 24‐h measurement (21.07 vs 4.93 dB) compared to commercial device.

Figure 5A shows the mobile application interface of the developed system, implemented to provide intuitive visualization of real‐time ECG waveforms measured via BLE, heart rate (HR) calculated from ECG R‐R peaks, and current activity status. HR and activity status are updated in real‐time to provide immediate correlation between user vital signs and physical activity. The details about HR detection are shown in the Experimental Section.

Not only the ECG and HR detection, but the developed device can also monitor static and dynamic activity status, including lying, sitting/standing, walking, and running, utilizing the embedded IMU sensor. Figure 5B presents tilt angle measurement results for verifying the static posture classification of the IMU sensor. The experimental device was fixed on a rotatable stand, and IMU sensor response was measured by rotating from 0 to 360 degrees in 30‐degree increments. Results showed correct classification of lying down between 330–30° and 150–210°, and sitting/standing between 60–120° and 240–300°. This systematic validation in controlled environments demonstrates that the developed IMU‐based posture classification can reliably determine user static posture in real‐world applications. Furthermore, the utilized IMU enables activity situation analysis. Using frequencies measured by the developed IMU, user activity status was defined as: sitting/standing (0–0.5 Hz), walking (1–1.5 Hz), and running (≥2 Hz). The signal with the maximum value among various signals occurring on arbitrary axes was utilized for analysis. Figure 5C shows IMU sensor frequency response characterization results using a vibration generator. By attaching the device to a vibration table and applying various frequency vibrations (0–3 Hz), activity status verification against frequency confirmed stable IMU sensor response in each frequency domain, reliably indicating defined activity status.

Figure 5D presents results obtained from performing various activities (lying, sitting/standing, walking, and running) for 1000 s while simultaneously wearing the development device and a commercial device. To objectively show the developed device performance, we also measure the biosignal utilizing a commercial electrode (Monitoring Electrode 2223H, 3 m Korea, Republic of Korea) and device (MEMO Patch 2, HUINNO, Republic of Korea) simultaneously (Figure S21). The middle ECG signal compares the development device (blue) and commercial device (gray) over the entire measurement period. ECG signals from the commercial device show crucial temporary amplitude increases, especially during walking and running, reflecting motion artifact effects caused by movement rather than signal quality improvement. In contrast, the developed device exhibits more stable ECG signal detection performance in all static and dynamic motion situations. Figure 5E shows magnified views of ECG signals measured by both devices in each activity state. For (i) lying down, (ii) sitting/standing, (iii) walking, and (iv) running, the developed device (blue) clearly and stably identifies PQRST waveforms under all conditions. Particularly noteworthy is signal shape retention even during walking and intense running. However, the commercial device (gray) shows crucial amplitude increases between walking and running while waveforms become more distorted and noisier, indicating the commercial device's susceptibility to motion artifacts in dynamic environments. The observed disparity in ECG signal quality between the devices has a direct influence on the accuracy and robustness of subsequent HR extraction. The extracted heart rate (HR) graphs exhibit increasing trends in both devices corresponding to changes in physical activity. However, the consistently stable HR profiles obtained from the development device, even during high‐motion activities such as running, demonstrate its strong resistance to motion artifacts and high reliability in accurate HR detection.

Furthermore, to evaluate the long‐term stability of the developed technology, 24‐h continuous measurement was performed. Figure 5F shows the ECG signal comparison at the measurement beginning (Initial) and end (24‐h). Signal shape and amplitude remain stable at both time points, while the commercial device shows signal quality degradation after extended periods.

Finally, a comparative summary of ECG detection performance between the developed device and the commercial device is presented. To further evaluate electrode performance under realistic long‐term and dynamic physiological conditions, ECG signals were measured during prolonged wear and under actual perspiration and chest expansion states. Measurements were conducted at three stages: immediately after application, after 4 h of wear, and after 8 h combined with controlled fast breathing and vigorous physical exercise to induce sweating. The results confirmed that P–QRS–T features remained distinct with stable amplitudes, verifying long‐term interfacial stability (Figure S22). In addition, ECG quality was analyzed during chest‐expansion motions such as deep breathing and stretching using electrodes with three serpentine geometries (135, 180, and 235°). As shown Figure S23, the optimized 235° design maintained consistent waveform fidelity and SNR values (4.98–13.8 dB), comparable to commercial gel electrodes. These findings demonstrate that the developed system preserves robust biosignal quality under sweating, stretching, and deep‐breathing conditions, confirming its suitability for long‐term wearable monitoring.

Prior to quantitative SNR evaluation, frequency‐domain analysis of ECG signals was conducted to verify the preservation of physiologically valid components. PSD analysis revealed that the developed system maintained higher power in the low‐frequency range (0–20 Hz), corresponding to characteristic ECG components (P, QRS, T waves), while effectively suppressing high‐frequency noise compared with the commercial device (Figure S24). These frequency‐domain results established the foundation for subsequent SNR quantification across various motion conditions.

Finally, a comparative summary of ECG detection performance between the developed device and the commercial device is presented. Figure 5G presents SNR analysis results for each activity state and long‐term measurement collectively in a radar chart format. The development device showed high SNR values of 22.12 dB for lying down, 26.19 dB for sitting/standing, 20.5 dB for walking, and 16.82 dB for running, outperforming the commercial device (4.98, 8.96, 6.18, and 13.8 dB, respectively) across all activities. Even after 24‐h long‐term measurements, the development device maintained 21.07 dB SNR while the commercial device significantly degraded to 4.93 dB, demonstrating the development device's suitability for continuous monitoring. As summarized Table S1, the developed electrode achieves a high SNR comparable to the best reported dry electrodes. Although some studies showed slightly higher SNR, those were obtained under limited laboratory settings, whereas our system ensures stable biosignal quality in realistic wearable conditions.

Additionally, complementary time‐domain analyses were performed to evaluate clinical stability metrics of the ECG signals, including ST‐segment and TP‐segment deviation (Figures S25–S27). The developed system exhibited significantly reduced baseline fluctuation and superior ST stability compared with the commercial device, confirming reliable waveform preservation under both static and dynamic conditions. Together with the PSD and SNR findings, these results validate that the proposed system ensures robust biosignal integrity suitable for long‐term, motion‐intensive wearable monitoring.

The developed technology was also utilized for electromyography (EMG) to verify system scalability (Figure S28). By attaching the developed system to the human leg soleus muscle and measuring EMG signals during standing, walking, and running, EMG signals were clearly distinguished between muscle contraction and relaxation in each activity state. Particularly, high‐amplitude signals reflecting strong muscle activity during running were reliably recorded, confirming that the developed electrodes can reliably measure bioelectrical signals during dynamic activities. This demonstrates that the system developed in this study extends beyond electrocardiogram measurement to various biosignal monitoring applications.

## 3 Conclusion

This study developed carbon black‐PDMS composite‐based dry electrodes addressing key challenges in long‐term vital sign monitoring. Through material composition optimization, 3D micro‐nano structures were formed on electrode surfaces, achieving excellent adhesion strength (15.68 kPa) and mechanical stiffness (216 kPa) below that of human skin. Notably, surface roughness changes with increasing carbon black concentration directly affected skin adhesion improvement, which, combined with stiffness optimization through PDMS base‐curing agent ratio adjustment, represented key factors in forming stable electrode‐skin interfaces. In accelerated aging tests at 85°C/85% RH, electrodes showed 3.51% adhesion strength increase and 16.87% impedance decrease after 36 h, indicating potential for improved performance during long‐term use. Beyond electrode material optimization, electrode interconnection structures were also optimized. Through skin deformation analysis and FEM simulation using 3D motion capture systems, an optimized serpentine structure (235°) was proposed, significantly reducing peeling stress (2.9 kPa) and enabling stable attachment during various human activities. This structural optimization also ensured electrical stability, with resistance change rates within 2% in x, y, and z directions.

Furthermore, a wireless wearable system was implemented and applied to developed electrodes to verify potential as real‐world wearable devices. First, verification confirmed that developed electrodes combined with commercially available electrocardiogram generators and compact hardware integrated with IMU can reliably measure electrocardiograms under experimental conditions (vibration, long‐term stability, water resistance, and repeated use). Finally, the feasibility evaluation of attaching developed electrodes and wearable devices directly to human bodies for vital sign and activity status measurement was conducted.

Results demonstrated that the developed device and mobile application integrated with real‐time activity status detection can acquire stable ECG signals with significantly higher SNR than commercial devices during various daily activities: lying (22.12 vs. 4.98 dB), sitting/standing (26.19 vs. 8.96 dB), walking (20.5 vs. 6.18 dB), and running (16.82 vs. 13.8 dB). Particularly, electrodes maintained excellent 21.07 dB SNR even after 24‐h continuous measurement (approximately 4‐fold better than the commercial device's 4.93 dB), demonstrating the developed electrode's suitability for prolonged active ECG monitoring.

This research improves the feasibility of wearable vital sign monitoring systems through systematic material and structural optimization. Repeated attachment–detachment testing up to 10,000 cycles demonstrated stabilized adhesion behavior without progressive degradation, supporting the mechanical robustness of the electrode–skin interface under laboratory conditions. Nevertheless, further validation across diverse skin types, varying hydration levels, and extreme perspiration environments will be important to fully assess inter‐subject impedance variability and real‐world robustness. In addition, while 24‐h continuous monitoring and accelerated aging tests suggest promising durability, extended multi‐day and clinical‐level evaluations are required to rigorously establish long‐term functional lifetime. Finally, although ECG and preliminary EMG feasibility were demonstrated, future efforts will focus on enhancing ultra‐low‐frequency interfacial impedance stability to enable reliable multimodal sensing, including electroencephalography (EEG) and electrodermal activity (EDA). These advancements will further position the developed platform for scalable, long‐term, and clinically relevant wearable bioelectronic applications.

Ultimately, this study establishes a novel yet accessible paradigm for high‐performance dry electrodes by successfully reconciling the trade‐off between fabrication simplicity and functional reliability. By elucidating the process‐structure‐property relationship of spontaneous micro‐texturization, we provide a scalable pathway to overcome the cost and complexity barriers of conventional micro‐structured electrodes. We believe that this motion‐artifact‐tolerant and skin‐compatible electrode platform will serve as a foundational technology for next‐generation digital healthcare systems, bridging the gap between laboratory research and ubiquitous clinical applications.

## 4 Experimental Section

### 4.1 Ethical Approval and Informed Consent

The in vivo human demonstrations, including electrophysiological monitoring (ECG, EMG), basic skin biocompatibility (erythema) tests, and skin displacement tracking using a 3D motion capture system, were performed solely on the authors of this study as non‐invasive self‐experimentation. The participating authors were fully aware of the experimental procedures and provided informed consent. Given the non‐invasive nature of these simple contact measurements, formal Institutional Review Board (IRB) approval was waived.

### 4.2 Statistical Analysis

Raw electrophysiological signals (e.g., ECG) acquired from the commercial and developed devices were normalized to account for baseline amplitude differences prior to further analysis. Frequency‐domain analysis, including Power Spectral Density (PSD) via Fast Fourier Transform (FFT), was conducted to evaluate the Signal‐to‐Noise Ratio (SNR) and signal fidelity. All quantitative data are expressed as the mean ± standard deviation (SD) obtained from at least three independent samples or measurements (*n* ≥ 3). For the in vitro cytotoxicity evaluation, statistical significance was determined using a two‐way analysis of variance (ANOVA) for the cell proliferation test and a one‐way ANOVA for the extract cytotoxicity test (ns: not significant, *P* > 0.05; **, *P* < 0.01). For other physical characterizations and signal processing, descriptive statistics were utilized. All data pre‐processing, statistical analysis, and graphical representations were performed using MATLAB R2022a (MathWorks), OriginPro 2024b (OriginLab).

### 4.3 Dry electrode Fabrication

Polydimethylsiloxane (PDMS, Sylgard 184, Dow Corning, USA) was prepared by mixing the base and curing agent at a ratio of 30:1, followed by mechanical stirring at 2,000 rpm for 1 min and de‐foaming at 2,200 rpm for 1 min using a planetary mixer (AR‐100, Thinky Corporation, Japan). After the base PDMS was homogeneously mixed and degassed, carbon black (CB, Vulcan XC 72R, Fuel Cell Store, USA) was added at 25 wt% (in PDMS), and the CB/PDMS composite was further mixed to achieve uniform dispersion.

Stretchable interconnectors were fabricated through a commercial flexible printed circuit board (FPCB) manufacturing service based on a custom‐designed layout. The FPCB consisted of a 50 µm thick copper (Cu) layer patterned into a serpentine structure with a 235° curvature angle. The detailed pattern design was implemented in‐house and outsourced to a FPCB fabrication vendor for production (JLCPCB, China).

The CB/PDMS composite was deposited onto the exposed electrode areas using screen printing through a 100 µm‐thick polyimide (PI) stencil mask. The printed electrodes were cured in a convection oven at 100°C for 180 min under ambient air conditions.

### 4.4 Finite Element Analysis of Skin–Electrode Interfacial Mechanics

Finite element simulations were performed using COMSOL Multiphysics to investigate the interfacial mechanical behavior between the micro‐textured CB/PDMS electrode and skin.

The skin layer was modeled as a linear elastic solid (Young's modulus = 1 MPa, Poisson's ratio = 0.48), representing soft biological tissue under moderate deformation conditions. The CB/PDMS composite electrode was modeled as a linear elastic material (Young's modulus = 200 kPa, Poisson's ratio = 0.49), reflecting its elastomeric characteristics. Geometric nonlinearity was enabled to account for large deformation and contact‐induced nonlinear effects.

Surface roughness (R_a_ = 0.1–1.0 µm) was parameterized using a sinusoidal surface profile defined as:

$$y\left(x\right)=A\sin\left(\frac{2\pi x}{\lambda }\right)$$

For a sinusoidal surface, the arithmetic mean roughness is given by:

$$\;R_{a}=\frac{1}{L}\;\int_{0}^{L}|y\left(x\right)|\mathrm{dx}=\frac{2A}{\pi }\;$$

Accordingly, the amplitude was calculated as:

$$A=\frac{\pi }{2}\;R_{a}$$

This formulation ensured a direct mathematical correspondence between experimentally measured roughness values and the geometric parameters implemented in the FEM model. The total model length was fixed at 900 µm for all simulations to enable direct comparison between different roughness conditions.

The skin–electrode interface was defined using a contact pair formulation with a Coulomb friction framework. The interfacial sliding resistance was incorporated based on experimentally measured lap shear adhesion values corresponding to each roughness condition. Specifically, the peak shear stress obtained from lap shear tests was used to constrain the allowable interfacial shear traction in the simulation, thereby establishing a direct linkage between experimental adhesion measurements and the FEM boundary conditions.

Two loading modes were applied to reflect practical deformation scenarios:

(i) a vertical displacement of 5 µm normal to the electrode surface, and (ii) a lateral displacement of 5 µm parallel to the skin surface.

The bottom boundary of the skin layer was fully fixed to eliminate rigid body motion. Loading was applied incrementally to ensure numerical stability.

A physics‐controlled triangular mesh with local refinement at the contact interface was employed to capture stress and displacement concentrations near micro‐asperities. Stationary nonlinear analysis was conducted using a fully coupled direct solver with geometric nonlinearity enabled.

Shear stress and displacement distributions at the skin–electrode interface were evaluated under both loading conditions. Static friction forces were calculated from interfacial reaction forces. Representative static friction values were obtained by averaging the peak values extracted from individual micro‐protrusion regions, enabling quantitative comparison of interfacial friction behavior as a function of surface roughness.

### 4.5 Wireless Measurement System

Biosignals acquired from developed electrodes are amplified and filtered by a commercial ECG chip (MAX30003, Analog Devices Inc., USA), then converted to digital signals via analog‐to‐digital conversion (ADC) in the microcontroller unit (MCU, STM32L432KCU6, STMicroelectronics, Switzerland). Converted signals are transmitted to mobile devices via Bluetooth module (BGM113, Silicon Labs, USA). Additionally, hardware was designed to monitor activity status by simultaneously acquiring user tilt and vibration information through an Inertial Measurement Unit (IMU, ICM‐20948, TDK InvenSense, USA) alongside ECG signals.

### 4.6 BPM and HR Detection

Heart rate detection was implemented using a cascaded digital signal processing approach. Raw ECG signals acquired from the developed electrodes were first amplified and filtered using a commercial ECG analog front‐end with a gain of 20 dB and a bandpass filter of 0.5–40 Hz to remove baseline drift and high‐frequency noise.

The filtered signals were digitized at a 125 Hz sampling rate using a 24‐bit analog‐to‐digital converter integrated in the microcontroller unit. Digital signal processing was performed using a multi‐stage filtering approach: (1) a 4th‐order Butterworth bandpass filter (5–15 Hz) to enhance QRS complex detection, (2) a derivative‐based peak detection algorithm to identify R‐peaks, and (3) adaptive thresholding to reject false peaks caused by noise or T‐wave interference.

Heart rate (HR) was calculated using the RR interval method:

HR (bpm) = 60 / (RR interval in seconds)

A sliding window of 10 consecutive RR intervals was used to calculate the average heart rate, with outlier rejection applied to intervals deviating more than 20% from the median value. The processed heart rate data was transmitted to a mobile application via Bluetooth Low Energy (BLE) for real‐time monitoring and data logging.

For activity‐based heart rate validation, the system incorporated a 9‐axis IMU to classify user activities (lying, sitting/standing, walking, running) based on acceleration magnitude and frequency characteristics. The IMU data was processed using a 125 Hz sampling rate with activity classification based on motion frequency thresholds: sitting/standing (0–0.5 Hz), walking (1–1.5 Hz), and running (≥2 Hz).

## Conflicts of Interest

The authors declare no conflict of interest.

## Supporting information




**Supporting File 1**: smll73136‐sup‐0001‐SuppMat.docx.

**Supporting File 2**: smll73136‐sup‐0002‐VideoS1.mp4.

## Data Availability

The data that support the findings of this study are available from the corresponding author upon reasonable request.
