# Supplementary material for: Micro‐Texturized and Ultra‐Soft Dry Electrode for Motion‐Artifact Tolerant and Long‐Term Stable Wearable Electrophysiological Monitoring
Source: Small. 2026 Mar 18;22(27):e14966. doi: 10.1002/smll.202514966 (PMC13173332; doi:10.1002/smll.202514966)
Supplement: Supplementary file 1 — Supporting File 1: smll73136‐sup‐0001‐SuppMat.docx. [file SMLL-22-e14966-s002.docx]

Supporting Information

**Micro-texturized and Ultra-soft Dry Electrode for Motion-artifact Tolerant and Long-term Stable Wearable Electrophysiological Monitoring**

Sang-Min Kim^1^, Hee Jeong Jang^2^, Ki-Hoon Kim^1^, Yeon-Jeong Hwang^1^, Yu Bin Lee^1^, Joonsoo Jeong^1,3^, Sunghoon Jung^4^, Eubin Jeong^4^, Dong-Wook Han^2^, Eui-Suk Sung^5,6^*, and Min-Ho Seo^1,3^*

^1^Department of Information Convergence Engineering, College of Information & Biomedical Engineering, Pusan National University, Busan, Republic of Korea

^2^Department of Cogno-Mechatronics Engineering, College of Nanoscience & Nanotechnology, Pusan National University, Busan, Republic of Korea

^3^School of Biomedical Convergence Engineering, College of Information & Biomedical Engineering, Pusan National University, Yangsan, Republic of Korea

^4^HUINNO Co., Ltd., Seoul, Republic of Korea

^5^Department of Otorhinolaryngology-Head and Neck Surgery, School of Medicine, Pusan National University, Yangsan, Republic of Korea

^6^Research Institute for Convergence of Biomedical Science and Technology, Pusan National University Yangsan Hospital, Yangsan, Republic of Korea

Corresponding authors: E.-S. Sung ([sunges@pusan.ac.kr](mailto:sunges@pusan.ac.kr)) & M.-H. Seo ([mhseo@pusan.ac.kr](mailto:mhseo@pusan.ac.kr))


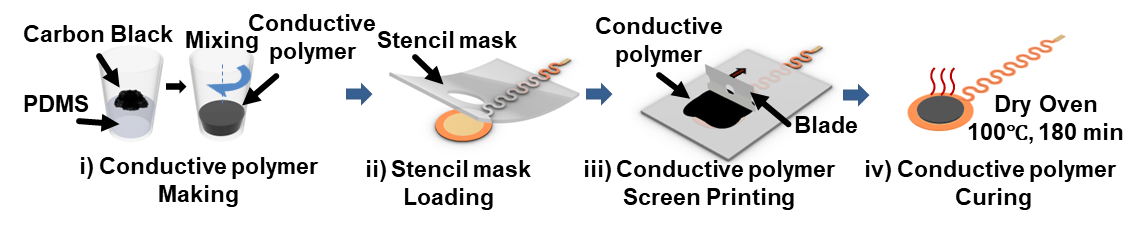


**Figure S1.** Schematic illustration of the electrode fabrication process: (i) preparation of CB/PDMS composite (25 wt% CB, 30:1 base-to-curing agent ratio), (ii-iii) screen-printing onto stretchable metal interconnector, and (iv) thermal curing at 100°C for 180 min.


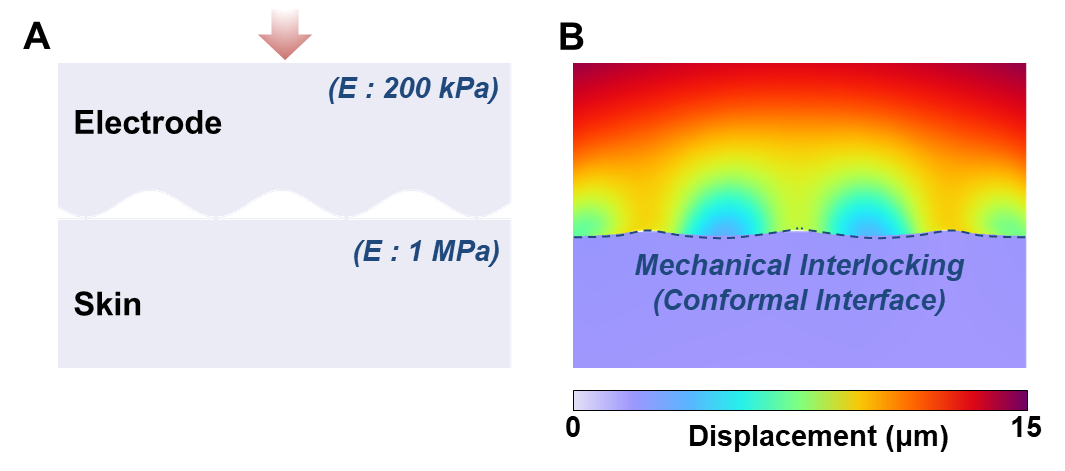
**Figure S2.** Finite element analysis of modulus-driven interfacial deformation and mechanical interlocking at the electrode–skin interface. **(A)** Schematic illustration of a compliant micro-textured electrode contacting a relatively stiffer skin substrate. **(B)** Finite element simulation showing deformation distribution under normal loading. Deformation is predominantly localized within the softer electrode layer, enabling conformal engagement of surface asperities and enhanced resistance to interfacial sliding.


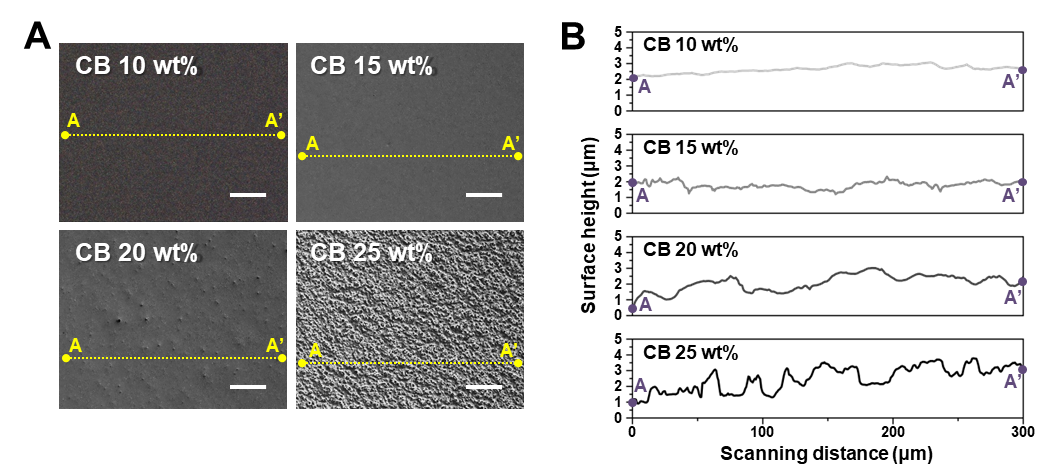


**Figure S3.** Surface morphology analysis of CB/PDMS composite films. **(A)** 3D profile images of PDMS (30:1) composites containing 10-25 wt% CB in PDMS with scanning paths (A-A') marked by yellow dotted lines (scale bars: 50 μm). **(B)** Surface height profiles measured along the 300 μm scan lines, demonstrating increased surface roughness with higher CB loading.


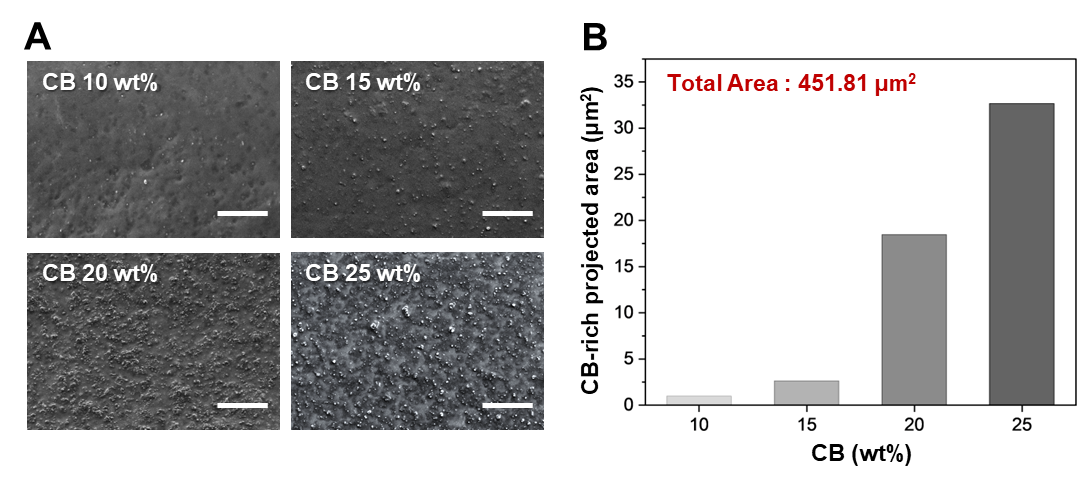


Figure S4. Quantitative cross-sectional SEM analysis of CB aggregation in CB/PDMS composites. (A) Cross-sectional SEM images of CB/PDMS composites with different CB loadings (10, 15, 20, and 25 wt%), where bright regions correspond to CB-rich domains (scale bars: 5 μm). (B) Quantified CB-rich area extracted from the SEM images using ImageJ, showing an increasing trend with CB concentration.


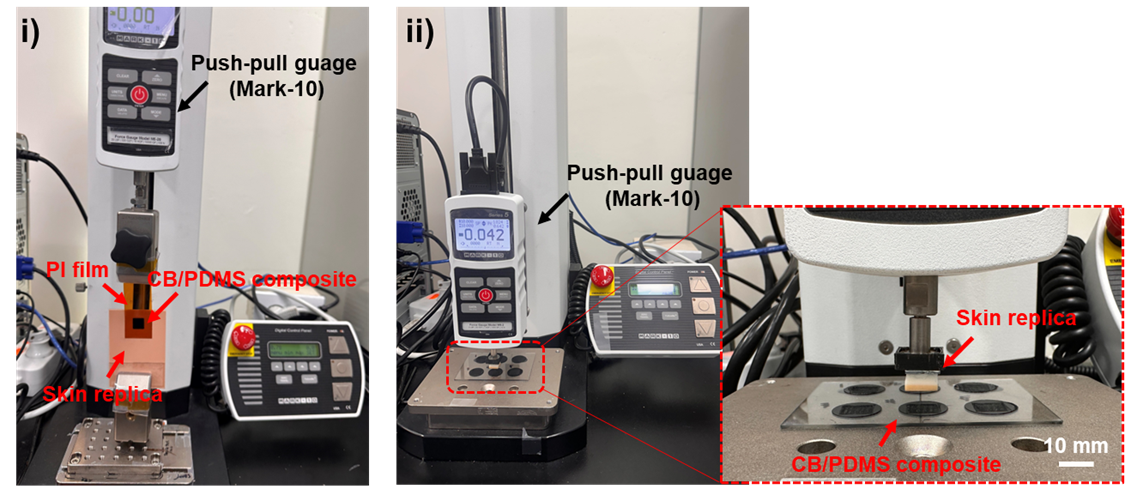


**Figure S5.** Experimental setup for adhesive strength measurement of CB/PDMS composite films using an automated push-pull gauge system (Mark-10, M5-2 & ESM303). i) Shear adhesion test configuration showing the PI film and CB/PDMS composite positioned on the skin replica (Suturing Skin Pad, HertzHardware, China) with tesile force application. ii) Normal adhesion test configuration with CB/PDMS composite samples positioned beneath the commercial artificial skin replica for vertical pull-off force measurement.


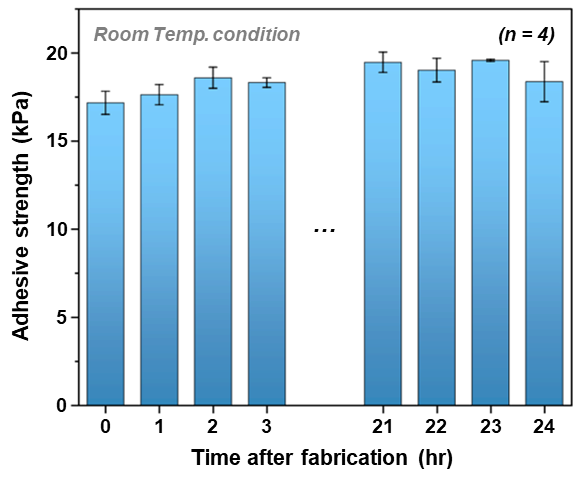
**Figure S6.** Time-dependent variation in adhesive strength of the CB/PDMS electrode after fabrication (*n = 4*). Electrodes were stored at ambient conditions before testing at the indicated time points.


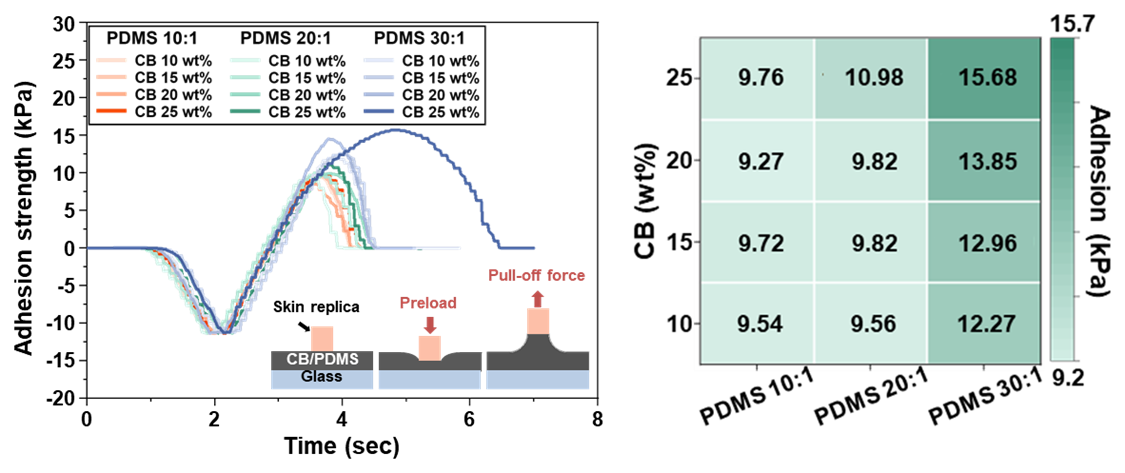


**Figure S7.** Adhesion strength measurements of CB/PDMS composites with varying PDMS ratios (10:1, 20:1, 30:1) and CB concentrations (10-25 wt% in PDMS). Higher CB loading demonstrates enhanced adhesion performance due to increased surface microstructuring and improved skin contact area.


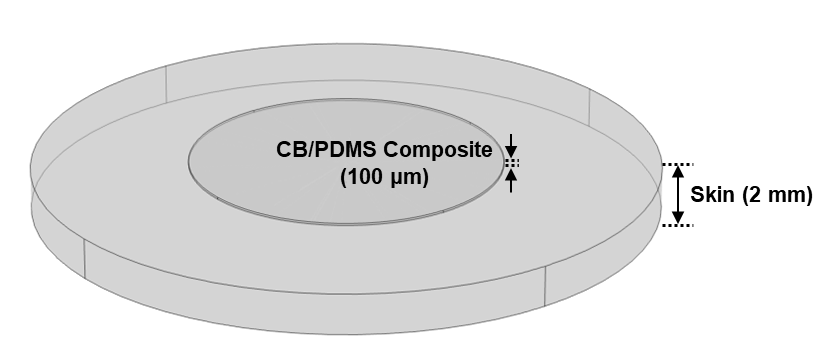


**Figure S8.** Model of the two-layered structure in COMSOL and FEM simulation conditions.


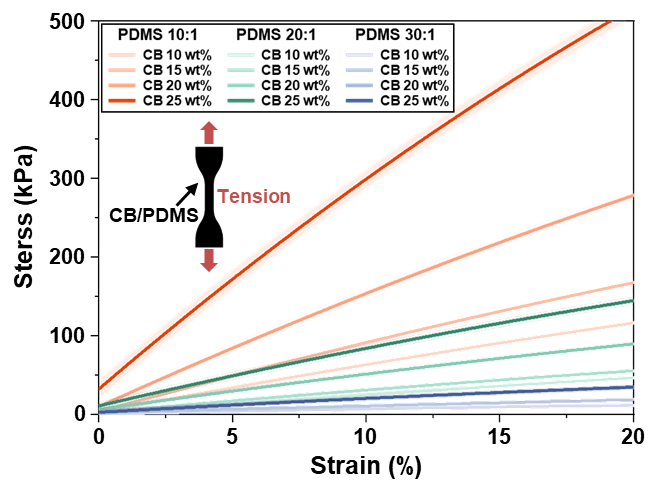


**Figure S9.** Tensile testing results of CB/PDMS composites with varying PDMS ratios (10:1, 20:1, 30:1) and CB concentrations (10-25 wt% in PDMS). Stiffness measurements at 20% strain were extracted to calculate electrode peeling stresses under skin tensile conditions, showing systematic increase in electrode stiffness with higher CB content and lower PDMS base ratios.


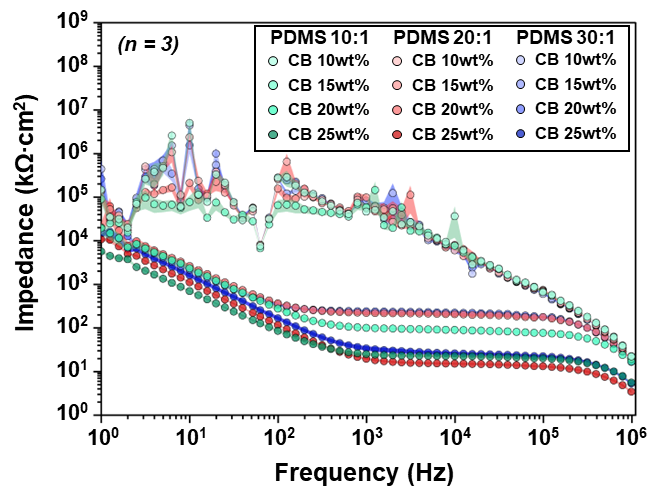


**Figure S10.** Electrochemical impedance characteristics of CB/PDMS composite electrodes with various material compositions. Impedance magnitude as a function of frequency (1 Hz to 1 MHz) for electrodes fabricated with different PDMS base-to-curing agent ratios (10:1, 20:1, and 30:1) and CB concentrations (10-25 wt% in PDMS). Measurements were conducted in PBS (pH 7.4) using a three-electrode configuration. Lower impedance values are achieved with higher CB concentrations, showing the most stable performance across the bioelectrical signal frequency range


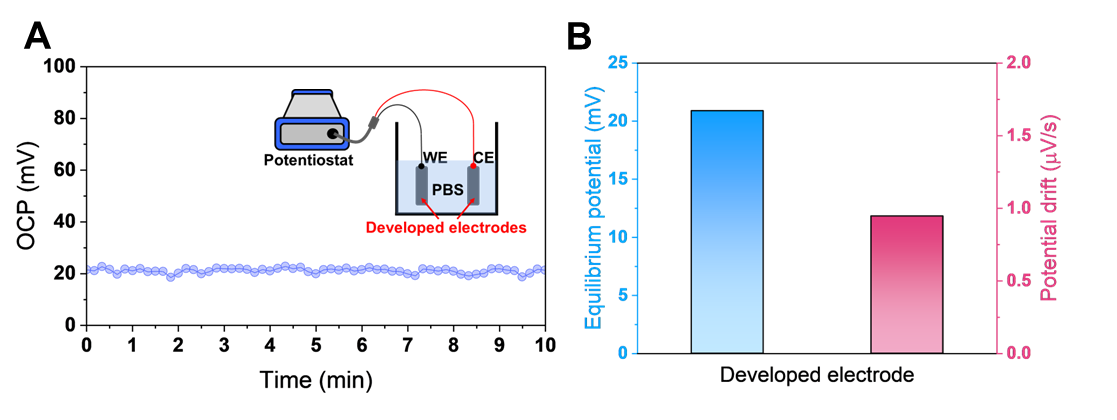


Figure S11. Electrochemical stability of the developed dry electrode. (A) Representative open-circuit potential (OCP) of the developed electrode measured for 10 min. (B) Corresponding equilibrium potential (20.89 mV) and potential drift (0.946 µV/s) extracted from the OCP data.


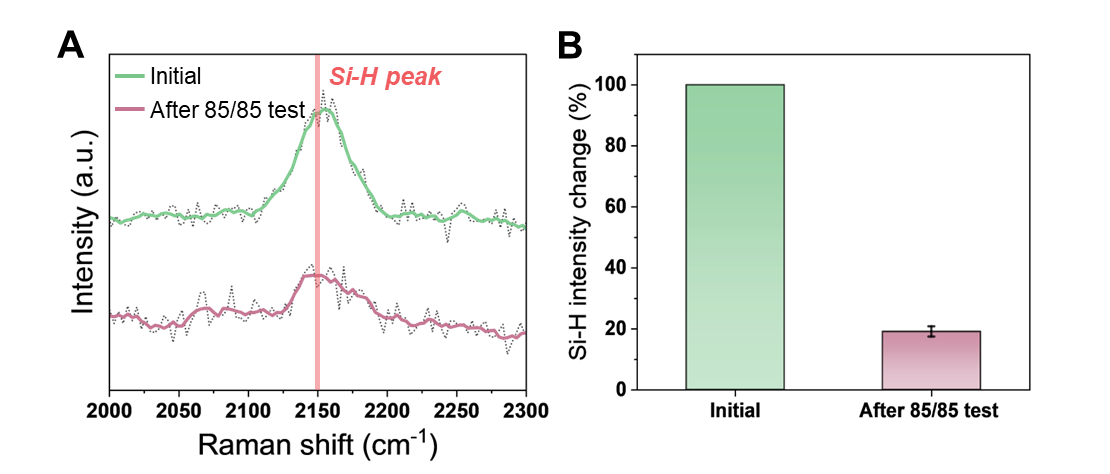


Figure S12. Raman spectroscopic results of Si–H elimination in the developed CB/PDMS composite after hygrothermal aging. (A) Raman spectra of the composite before and after exposure to 85 °C/85% RH, highlighting the Si–H stretching vibration (~2150 cm⁻¹). (B) Quantified change in Si–H peak intensity, showing an 80.9% reduction after aging, indicating consumption of residual Si–H groups via post-curing and moisture-assisted reactions.


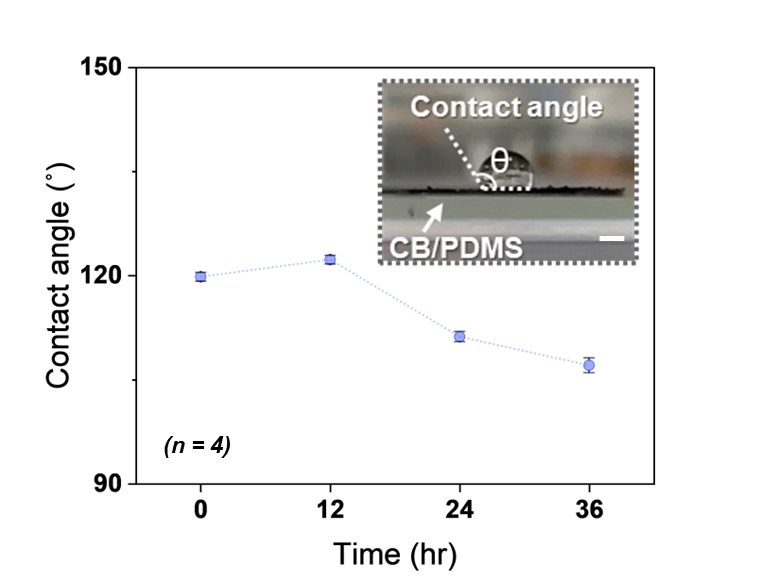


Figure S13. Time-dependent water contact angle of the CB/PDMS composite during hygrothermal aging. Static water contact angle measured on the CB/PDMS surface after exposure to 85 °C/85% RH for different aging durations (*n = 4*). The gradual decrease in contact angle indicates enhanced surface hydrophilicity induced by moisture adsorption and chemical surface evolution. The inset shows a representative optical image used for contact angle determination (scale bar: 1 mm).


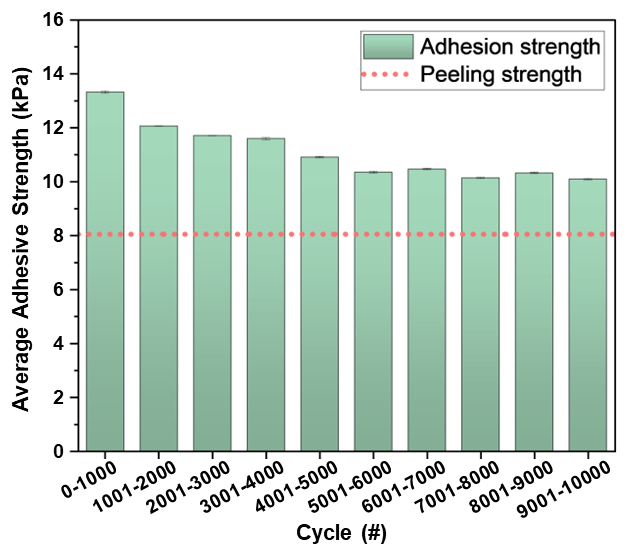


**Figure S14.** Reusability of the CB/PDMS-based dry adhesive electrode over 10,000 peel–reattach cycles. Each point represents the average adhesive strength measured over every 1,000-cycle interval. A pink dashed line indicates the threshold strength (8.054 kPa) required for stable skin adhesion.


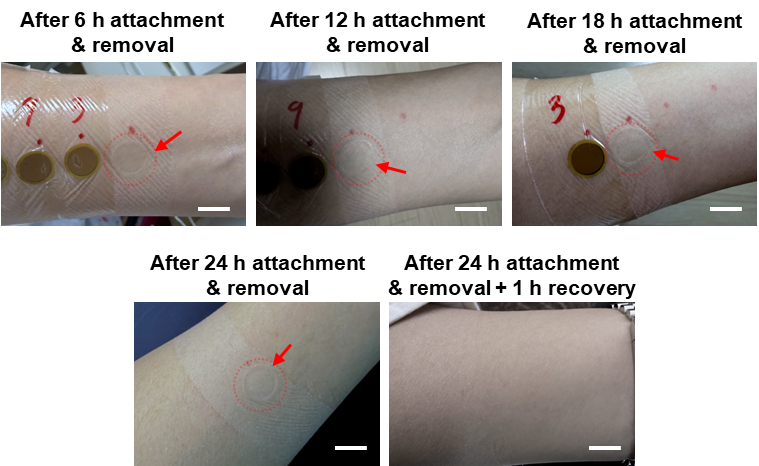


Figure S15. Preliminary skin compatibility test of the developed dry electrode. Representative photographs of the forearm skin after simultaneous attachment of multiple electrode patches followed by sequential removal at 6 h intervals. No visible signs of skin irritation or erythema were observed after up to 24 h of skin contact. Red arrows indicate the electrode attachment sites (scale bar: 10 mm).


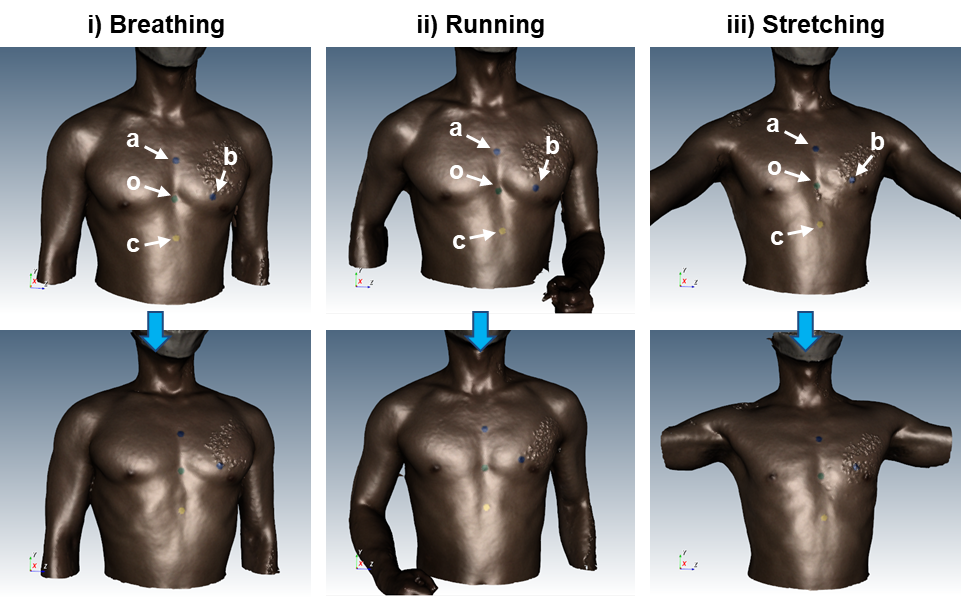


**Figure S16.** 3D motion capture analysis of skin deformation during human activities. Visualization of skin surface displacement at electrode attachment points (a, b, c, and o) during (i) breathing, (ii) running, and (iii) stretching movements. The upper and lower images show the initial and deformed states for each activity, respectively. Quantitative analysis of mechanical deformation during typical human motion provides key design parameters for serpentine electrode optimization.


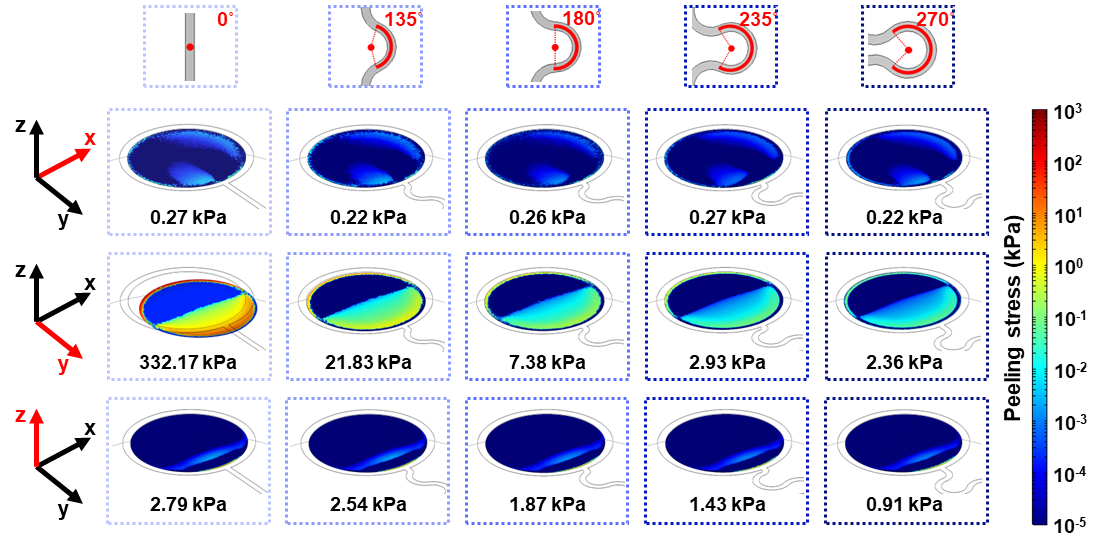


**Figure S17.** Detailed finite element analysis of peeling stress in serpentine electrodes under triaxial displacement. FEM simulation results show peeling stress distribution for serpentine angles from 0° to 270° under 30 mm displacement in x, y, and z directions, with maximum peeling stress values indicated below each electrode geometry. Results demonstrate significant stress reduction with increasing serpentine angle, particularly in the y-direction, with the optimized 235° structure achieving peeling stresses below the electrode adhesion threshold (≈16 kPa) in all directions.


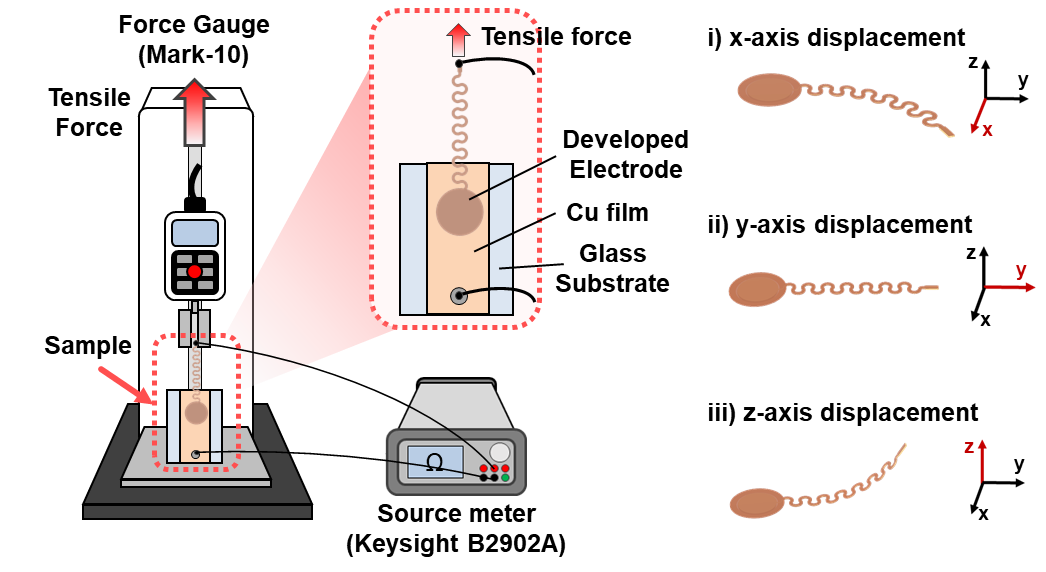


**Figure S18.** Experimental setup for electro-mechanical stability evaluation of serpentine electrodes. Schematic illustration of the triaxial tensile testing system using a Mark-10 force gauge and Keysight B2902A source meter for simultaneous mechanical and electrical characterization. The developed electrode with serpentine interconnector structure is subjected to controlled displacement in (i) x-axis, (ii) y-axis, and (iii) z-axis directions while monitoring resistance changes to evaluate electro-mechanical stability under various deformation conditions.


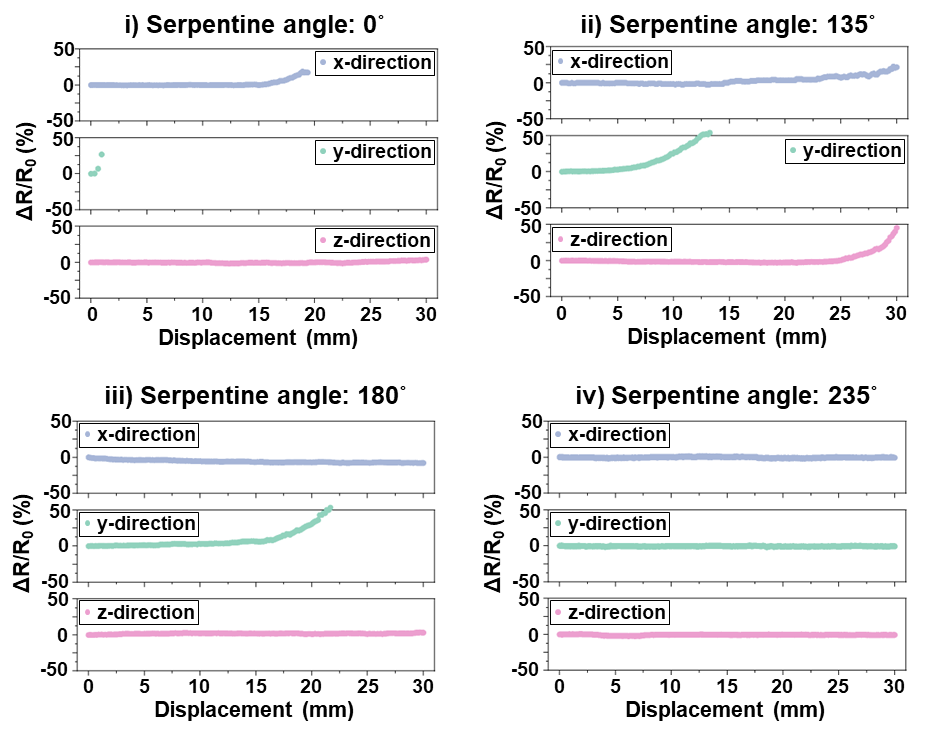


**Figure S19.** Electro-mechanical characterization of serpentine electrodes under triaxial mechanical deformation. Relative resistance change (ΔR/R₀) during triaxial tensile testing for serpentine angles of i) 0°, ii) 135°, iii) 180°, and iv) 235° in x- (blue), y- (green), and z-directions (pink). Progressive improvement in electrical stability with increasing serpentine curvature demonstrates structure-property relationships, with optimized 235° geometry achieving <2.5% resistance variation compared to >25% for straight interconnects under equivalent mechanical loading.

**
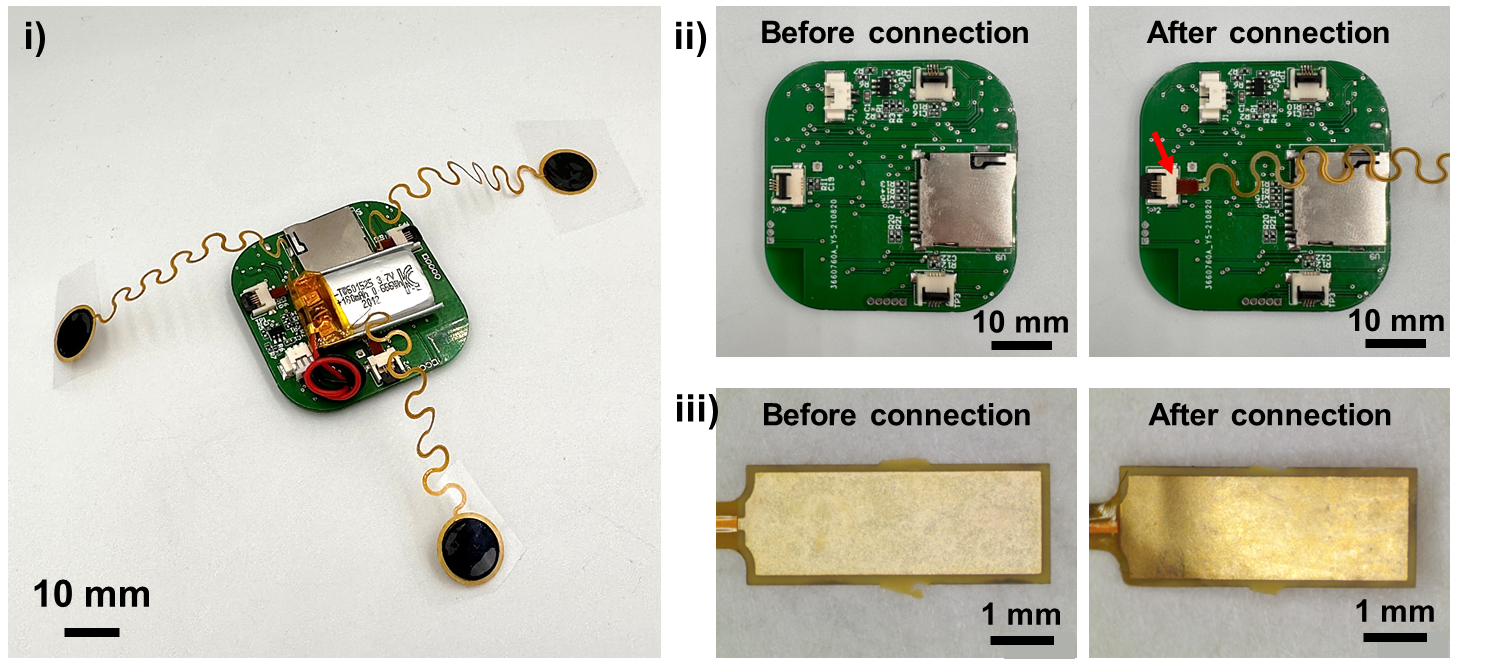
**

**Figure S20.** Photographs of the integrated wearable system with flexible electrodes. (i) Full system view showing serpentine interconnects of dry electrodes connected to the readout circuit. (ii) Optical images of the PCB before and after connecting the developed electrodes. The red arrow indicates the connected region. (iii) Magnified views of the flexible PCB connector region before and after connection, confirming secure insertion without visible mechanical damage.


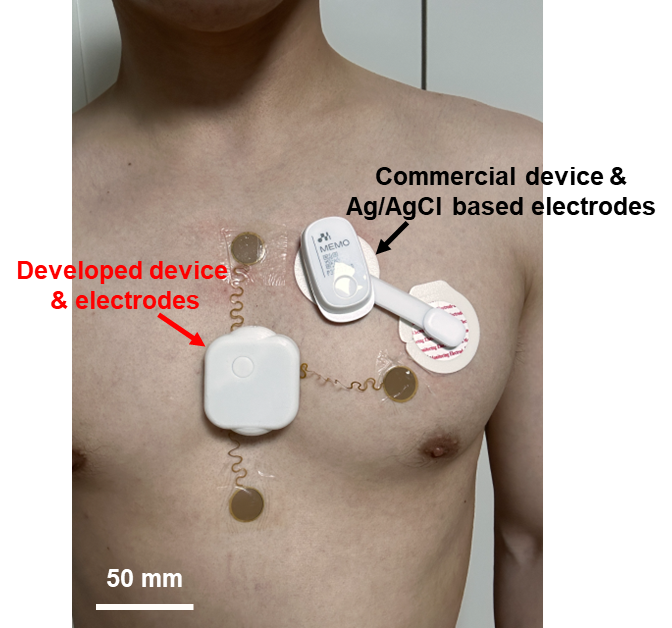


**Figure S21.** Photographic comparison of the developed wearable ECG device and electrodes (left) with a commercial ECG system, MEMO Patch 2 (HUINNO, Republic of Korea), using Ag/AgCl gel electrodes (Monitoring Electrode 2223H, 3M Korea) (right), simultaneously attached to a human subject for real-time biosignal monitoring. The developed system integrates flexible CB/PDMS composite electrodes with a miniaturized wireless module, while the commercial device employs rigid snap-type gel electrodes. This setup was used to objectively compare signal quality and motion artifact suppression across various activity conditions..


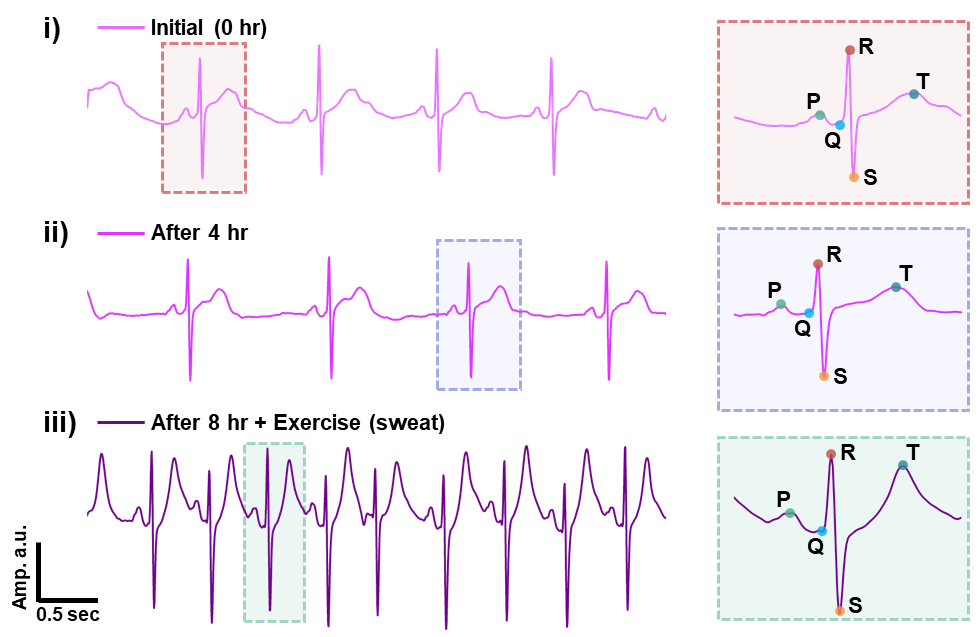


**Figure S22.** ECG signal stability during long-term wear with physical activity and sweating (i) ECG signals recorded immediately after developed system attachment (initial), (ii) after 4 hours of continuous wear, and (iii) after 8 hours including physical activity with heavy sweating. Right panels show zoomed-in PQRST segments, confirming stable ECG morphology and signal quality despite extended wear and challenging real-world conditions such as sweat and movement.


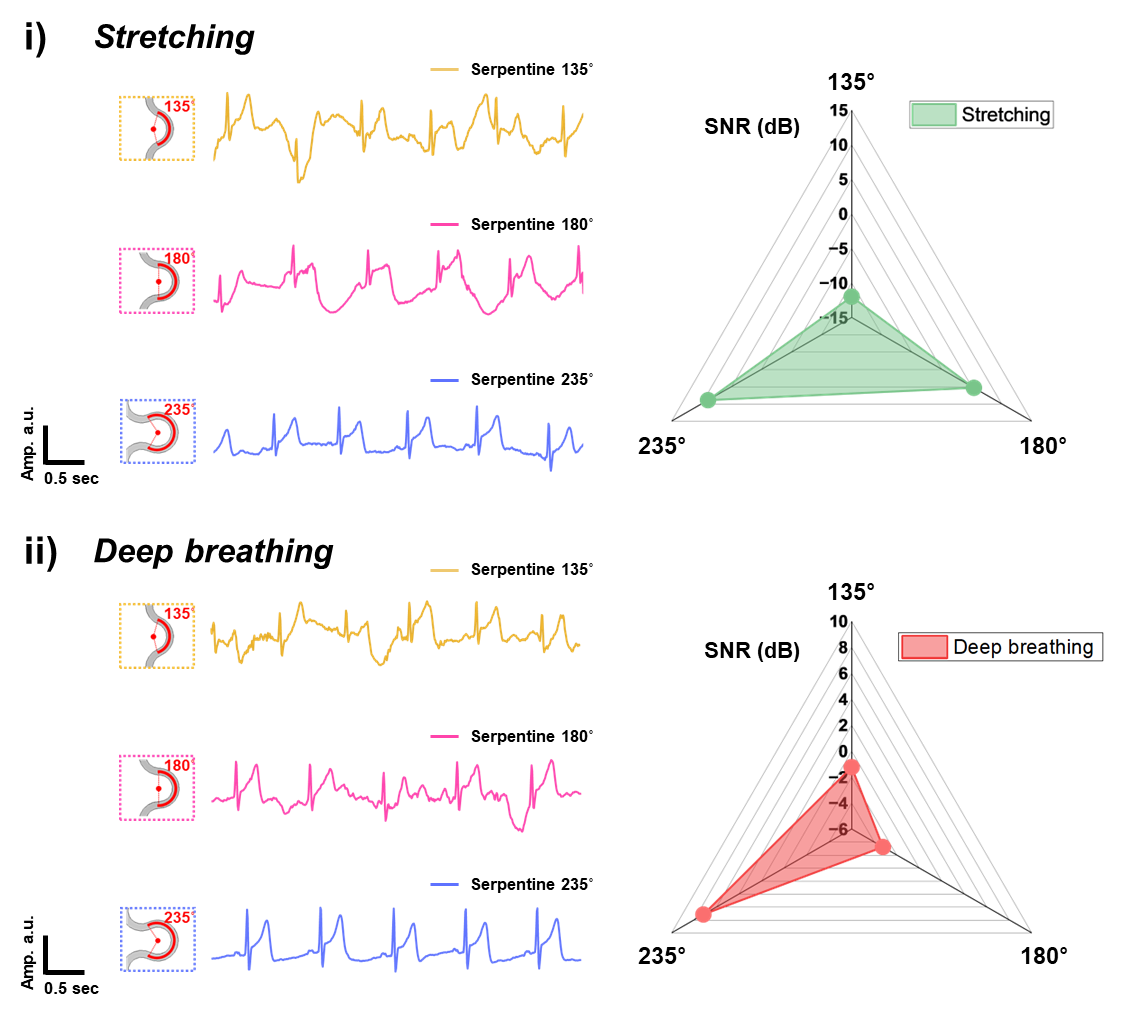


**Figure S23.** ECG signal analysis under realistic wearable conditions. (i) ECG waveforms recorded during arm stretching using electrodes with serpentine interconnects of different angles (135°, 180°, 235°). Increased curvature improves signal stability and SNR. (ii) ECG signals measured during deep breathing, simulating upper body motion. The 235° design shows superior artifact resistance.Radar plots summarize SNR values, highlighting the impact of interconnect geometry on motion robustness.


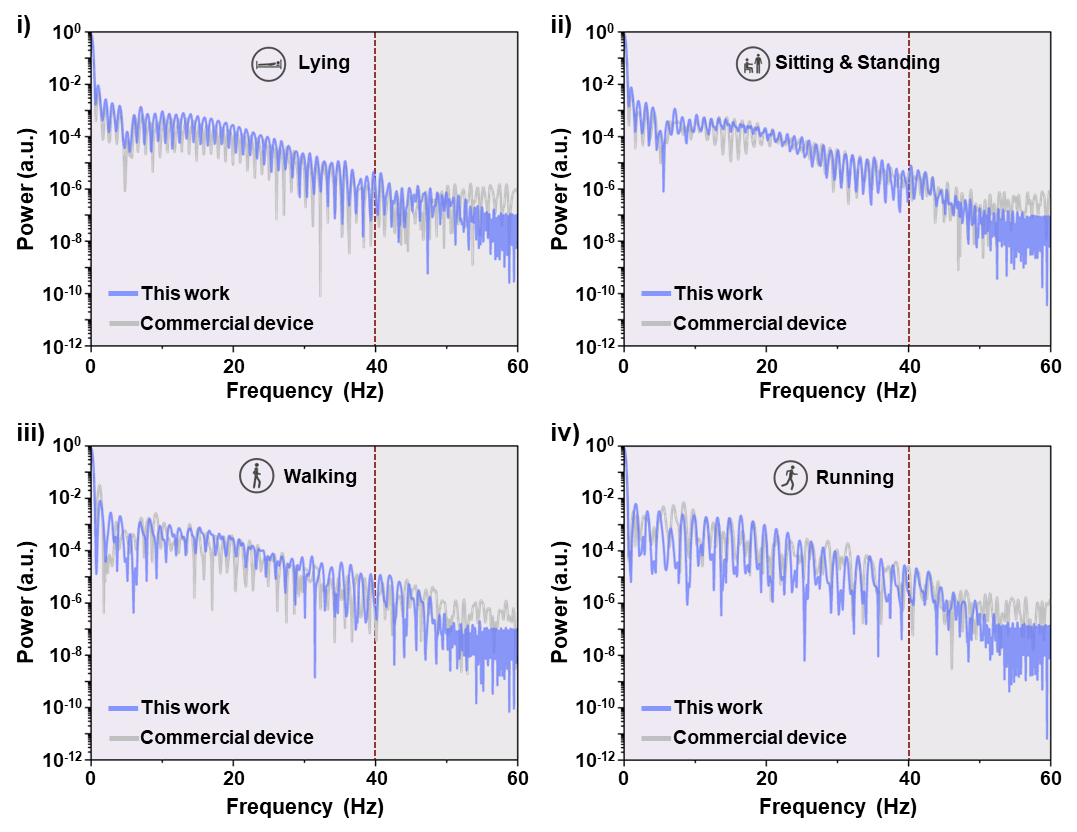


**Figure S24.** Power spectral density (PSD) comparison of ECG signals between the developed system and a commercial device with Ag/AgCl electrodes under different motion conditions. Representative PSD spectra were obtained from normalized ECG signals recorded during (i) lying, (ii) sitting & standing, (iii) walking, and (iv) running. The developed system (blue) preserved higher power in the low-frequency range (0–20 Hz) corresponding to physiological ECG components (P, QRS, T waves), while suppressing high-frequency noise components compared to the commercial device (gray). Across all activity conditions, the PSD profile of the developed system remained consistent, demonstrating superior resistance to motion-induced artifacts and improved signal fidelity during dynamic body movements.


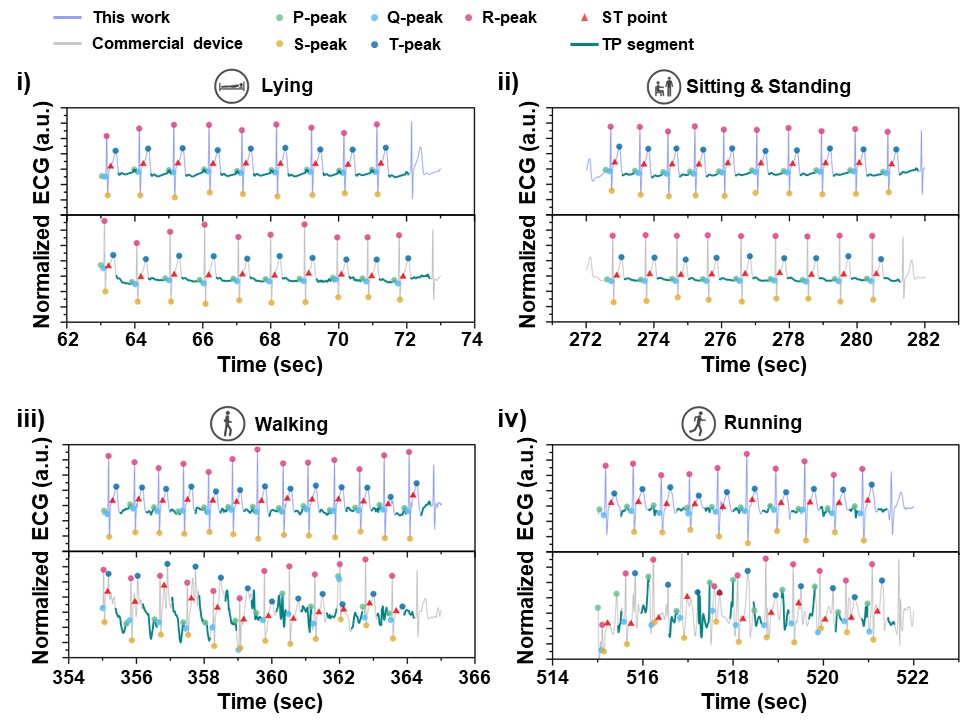


**Figure S25.** Peak detection and segmentation of ECG signals for ST-segment stability and TP-deviation analysis. Representative normalized ECG waveforms obtained from the developed system (blue) and a commercial device with Ag/AgCl electrode (gray) under four different motion states: (i) lying, (ii) sitting & standing, (iii) walking, and (iv) running. Detected characteristic points—P (green), Q (blue), R (pink), S (orange), T (cyan)—and ST points (red triangles) are annotated for each beat.


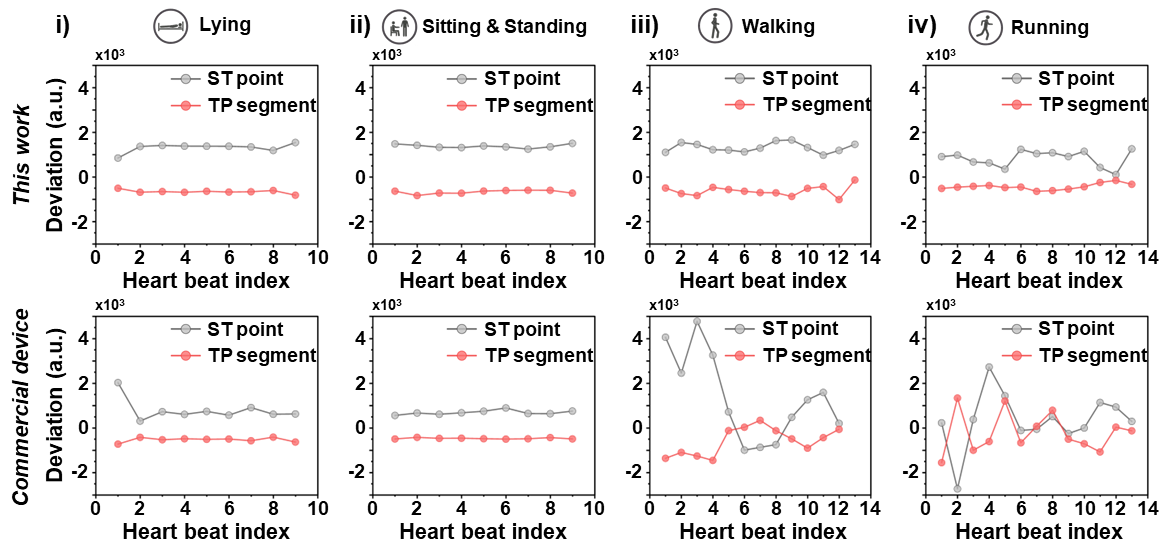


**Figure S26.** Comparison of ST-segment and TP-segment deviation stability between the developed system and a commercial device with Ag/AgCl electrodes under various motion states. Heartbeat-wise deviation plots of ST-segment (top row) and TP-segment (bottom row) for the developed system (red) and the commercial device (gray) under four motion conditions: (i) lying, (ii) sitting & standing, (iii) walking, and (iv) running. The developed system exhibits significantly smaller deviations across successive heartbeats in both ST and TP segments, demonstrating enhanced baseline stability and reduced drift even during motion.


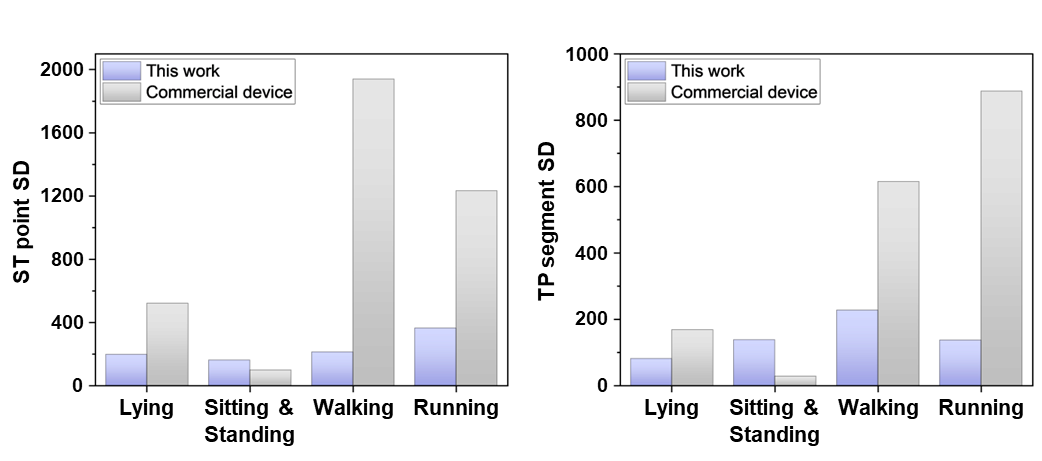


**Figure S27.** Quantitative comparison of ST-segment and TP-segment deviation stability between the developed system and a commercial device with Ag/AgCl electrodes. Standard deviations of (A) ST-segment deviation and (B) TP-segment deviation extracted from ECG recordings under four motion states — (i) lying, (ii) sitting & standing, (iii) walking, and (iv) running. Under static postures (lying and sitting & standing), both systems showed comparable stability, as expected due to minimal motion interference. However, under dynamic conditions (walking and running), the developed system (blue) exhibited substantially smaller variations than the commercial device (gray), confirming enhanced baseline stability and reduced motion-induced drift.


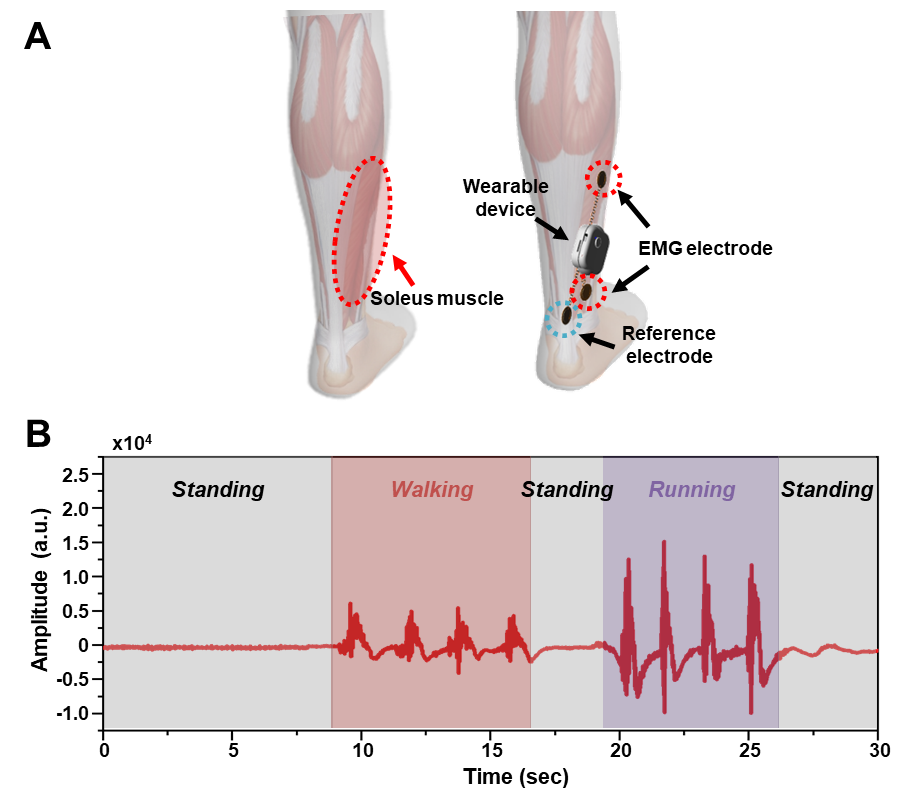


**Figure S28.** Electromyography (EMG) signal measurement using the developed electrode system. **(A)** Electrode placement on the human leg for soleus muscle EMG monitoring, showing the positioning of EMG electrodes, reference electrode, and wearable device. **(B)** Real-time EMG signals recorded during different physical activities including standing, walking, and running. The developed electrodes successfully distinguish muscle contraction and relaxation states, with high-amplitude signals clearly observed during running activity, demonstrating the system's capability for reliable bioelectrical signal acquisition across various movement conditions.


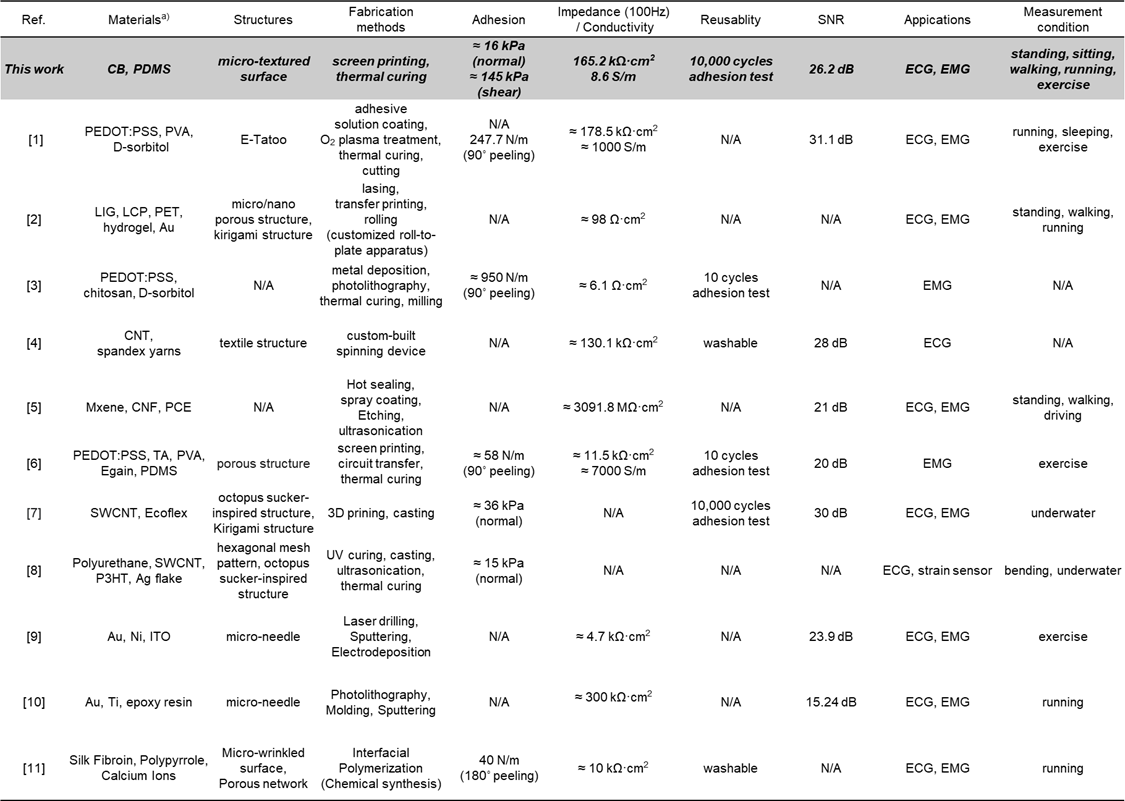

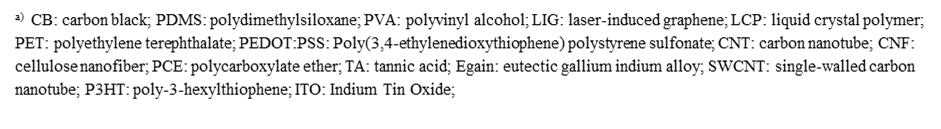
**Table S1.** Comparison of performance metrics of structurally engineered dry electrodes.

REFERENCES

1. J. H. Shin, J. Y. Choi, K. June, H. Choi, T. Kim, Polymeric Conductive Adhesive-Based Ultrathin Epidermal Electrodes for Long-Term Monitoring of Electrophysiological Signals. *Adv. Mater.* 2024, 36, 2313157. https://doi.org/10.1002/adma.202313157
2. R. Park, D. H. Lee et al. Laser-Assisted Structuring of Graphene Films with Biocompatible Liquid Crystal Polymer for Skin/Brain-Interfaced Electrodes. *Adv. Healthcare Mater.* 2024, 13 (3), 2301753. https://doi.org/10.1002/adhm.202301753
3. Z. Zhao, H. Yu, D. J. Wisniewski, C. Cea, L. Ma, E. M. Trautmann, M. M. Churchland, J. N. Gelinas, D. Khodagholy, Formation of Anisotropic Conducting Interlayer for High-Resolution Epidermal Electromyography Using Mixed-Conducting Particulate Composite. *Adv. Sci.* 2024, 11, 2308014. https://doi.org/10.1002/advs.202308014
4. Hossain, M.M., Li, B.M., Sennik, B. et al. Adhesive free, conformable and washable carbon nanotube fabric electrodes for biosensing. *npj Flex. Electron.* 2022, 6, 97. https://doi.org/10.1038/s41528-022-00230-3
5. Lee, S., Ho, D.H., Jekal, J. et al. Fabric-based lamina emergent MXene-based electrode for electrophysiological monitoring. *Nat. Commun.* 2024, 15, 5974. https://doi.org/10.1038/s41467-024-49939-x
6. Yang, S., Cheng, J., Shang, J. et al. Stretchable surface electromyography electrode array patch for tendon location and muscle injury prevention. *Nat. Commun.* 2023, 14, 6494. https://doi.org/10.1038/s41467-023-42149-x
7. G. R. Kang et al. Robustly Repeatable, Permeable, and Multi-Axially Stretchable, Adhesive Bioelectronics With Super-adaptive Conductive Suction Cups for Continuously Deformable Biosurfaces. *Adv. Sci.* 2025, 12, 2500346. https://doi.org/10.1002/advs.202500346
8. Hyeongho Min et al. Highly Air/Water-Permeable Hierarchical Mesh Architectures for Stretchable Underwater Electronic Skin Patches. *ACS Appl. Mater. Interfaces* 2020, 12 (12), 14425-14432. https:// dx.doi.org/10.1021/acsami.9b23400
9. Chuying Sun et al. Noninvasive, Ultrathin, Flexible Microneedle Electrodes for Accurate and Long-Term Biopotential Monitoring. *ACS Applied Materials & Interfaces* 2025 17 (47), 64077-64086. https://doi.org/10.1021/acsami.5c13122
10. Hou et al. Miura-ori structured flexible microneedle array electrode for biosignal recording. *Microsystems & Nanoengineering* 2021 7:53. https://doi.org/10.1038/s41378-021-00259-w
11. Hui Yang et al. Adhesive Biocomposite Electrodes on Sweaty Skin for Long-Term Continuous Electrophysiological Monitoring. *ACS Materials Lett.* 2020,2,478−484. https://dx.doi.org/10.1021/acsmaterialslett.0c00085
